# *Coreopsis tinctoria* Nutt. Polyphenols Ameliorate CUMS‐Induced Depressive Behaviors and Hippocampal Neuronal Damage in Mice

**DOI:** 10.1002/fsn3.72480

**Published:** 2026-10-16

**Authors:** Haixia Han, Yujing Feng, Zhen Wang, Rui Ma, Jingjing Sun, Xiuli Yang, Yue Cui, Juan Wang, Mingxi Jia

**Affiliations:** ^1^ College of Food Science and Pharmacy Xinjiang Agricultural University Urumqi China; ^2^ Xinjiang Key Laboratory of Postharvest Fruit Science and Technology Urumqi China; ^3^ College of Modern Industry in Chinese Herbal Medicine Xinjiang Agricultural University Urumqi China; ^4^ Department of Anesthesiology Shanghai Punan Hospital of Pudong New District Shanghai China

**Keywords:** CCT polyphenols extract (CCTE), chronic unpredictable mild stress (CUMS), depression, HPA axis, NLRP3/NF‐κB pathway

## Abstract

Chronic stress‐induced mood disturbances have become a prominent public health concern, and dietary polyphenols are emerging as natural nutritional strategies for mood health management. The capitula of *Coreopsis tinctoria* Nutt. (CCT) are edible flowers rich in polyphenols, but their mood‐supporting efficacy remains unclear. This study investigated the effects, mechanisms, and key bioactive constituents of CCT polyphenol extract (CCTE) in a chronic unpredictable mild stress (CUMS) mouse model. High‐dose CCTE (1 g/kg) restored sucrose preference from 30.9% ± 4.1% in CUMS mice to 52.0% ± 5.5%, increased week‐6 weight gain from 4.7% ± 2.4% to 38.8% ± 5.9% (*p* < 0.001), and restored open‐field rearing, central‐area crossings, and residence time to fluoxetine‐comparable levels, indicating dose‐dependent attenuation of CUMS‐induced depressive‐like behaviors. It also ameliorated hippocampal neuronal damage, corrected the CUMS‐induced 36.7% reduction in serum 5‐HT and 44.8% elevation in 5‐HIAA, attenuated hypothalamic–pituitary–adrenal (HPA) hyperactivity, particularly reducing elevated CRH, ACTH, and CORT levels, alleviated oxidative stress, and suppressed NLRP3 inflammasome–driven neuroinflammation via NLRP3/NF‐κB downregulation. Three flavonoid aglycones—okanin (OKA), isookanin (ISO), taxifolin (TAX)—crossed the blood–brain barrier (free *C*_max_ 28.45 ± 10.9, 32.74 ± 27.3, 32.04 ± 6.9 ng/g; *T*_max_ 20–45 min) and inhibited monoamine oxidase A (MAO‐A; IC_50_ 38.66 ± 2.45, 48.23 ± 3.12, 47.28 ± 2.98 μmol/L); their equimolar mixture acted synergistically (IC_50_ 18.79 ± 1.67 μmol/L; pargyline 8.71 ± 1.21 μmol/L, *p* < 0.001). Thus, CCTE ameliorates stress‐induced mood disturbances through multi‐target regulation and shows potential as a functional food ingredient.

## Graphical abstract

The capitula of *Coreopsis tinctoria* Nutt. polyphenol extract (CCTE) attenuate CUMS‐induced depressive‐like behaviors and hippocampal neuronal damage in mice. Three flavonoid aglycones—okanin, isookanin, and taxifolin—cross the blood–brain barrier and synergistically inhibit MAO‐A, restoring serotonergic tone; concurrently, CCTE normalizes HPA‐axis hyperactivity, alleviates oxidative stress, and suppresses NLRP3/NF‐κB‐driven neuroinflammation. Thus, *C. tinctoria* polyphenols act through multi‐target regulation and represent a promising functional‐food strategy for mood health.

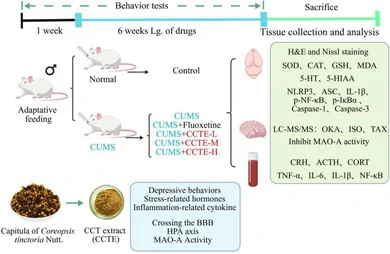

**Abbreviations**
5‐HIAA: 5‐hydroxyindoleacetic acid
5‐HT: 5‐hydroxytryptamine
ACTH: adrenocorticotropic hormone
BBB: blood–brain barrier
CA: chlorogenic acid
CAT: catalase
CCT: capitula of *Coreopsis tinctoria* Nutt.
CCTE: CCT polyphenols extract
CORT: corticosterone
CRH: corticotropin‐releasing hormone
CUMS: chronic unpredictable mild stress
FCA: 4,5‐di‐O‐caffeoylquinic acid
FDG: 5,7,3′,5′‐tetrahydroxyflavanone‐7‐O‐β‐D‐glucopyranoside
FMR: flavanomarein
GSH: glutathione
HPA: hypothalamic–pituitary–adrenal
IOD: integrated optical density
ISO: isookanin
MAO: monoamine oxidase
MAO‐A: monoamine oxidase A
MDA: malondialdehyde
MR: marein
NF‐κB: nuclear factor kappa‐B
NLRP3: NOD‐like receptor protein 3
OKA: okanin
PBS: phosphate‐buffered saline
p‐NF‐κB: phosphorylated nuclear factor kappa‐B
QDG: quercetagetin‐7‐O‐β‐D‐glucopyranoside
SERT: serotonin transporter
SOD: superoxide dismutase
SSRIs: selective serotonin reuptake inhibitors
TAX: taxifolin
TCA: 3,5‐di‐O‐caffeoylquinic acid
TDG: taxifolin‐7‐O‐β‐D‐glucopyranoside
TNF‐α: tumor necrosis factor‐α

## 1 Introduction

Chronic stress‐induced mood disturbances have become a prevalent health issue affecting general populations, and nutritional interventions based on natural food ingredients are increasingly recognized as a safe and sustainable strategy for daily mood health management (Yin et al. 2024). Accumulating evidence indicates that the pathogenesis of mood dysregulation involves multiple interconnected physiological disruptions, including hyperactivity of the hypothalamic–pituitary–adrenal (HPA) axis, chronic low‐grade neuroinflammation, oxidative stress, impaired neurotrophic support, and monoamine neurotransmitter imbalance, which together form a complex multisystem regulatory network (Ding et al. 2024; Wang et al. 2021). Conventional pharmaceutical interventions, while effective for clinical treatment, are associated with limitations such as side effects and restricted applicability for daily preventive use. Accordingly, developing natural functional food ingredients with multi‐target nutritional regulatory effects, good tolerability, and suitability for long‐term dietary intake has become an important research direction in food science and human nutrition (Duman et al. 2016).

In this context, dietary polyphenols from edible plants have emerged as a valuable class of bioactive ingredients for mood health support, owing to their multi‐component composition, multi‐target biological activities, and high safety as food components (Noori et al. 2022). Studies have shown that plant‐derived polyphenol extracts not only modulate monoaminergic systems but also exert anti‐inflammatory, antioxidant, neuroprotective, and HPA‐axis‐regulating effects. These properties collectively endow them with “multi‐component, multi‐target, and multi‐pathway” nutritional functional potential, offering distinct advantages in daily management of stress‐related mood disturbances (Behl et al. 2022; Rog et al. 2026).

*Coreopsis tinctoria* Nutt. is a plant species belonging to the genus Coreopsis in the Asteraceae family. The capitula of *C. tinctoria* (CCT) have been used in food processing and traditional medicine in North America, Europe and Asia (Mi et al. 2023). Studies have indicated that CCT are rich in polyphenolic bioactive constituents, including flavonoids and phenolic acids (Ma et al. 2016; Wang et al. 2026). In our previous work, we isolated and identified 11 major polyphenols from CCTE: marein (MR), flavanomarein (FMR), taxifolin‐7‐O‐β‐D‐glucopyranoside (TDG), quercetagetin‐7‐O‐β‐D‐glucopyranoside (QDG), okanin (OKA), isookanin (ISO), 5,7,3′,5′‐tetrahydroxyflavanone‐7‐O‐β‐D‐glucopyranoside (FDG), taxifolin (TAX), chlorogenic acid (CA), 3,5‐di‐O‐caffeoylquinic acid (TCA), and 4,5‐di‐O‐caffeoylquinic acid (FCA), and demonstrated their significant in vitro antioxidant activity (Ma et al. 2016). Further studies using mouse behavioral despair models (the tail suspension test and the forced swimming test) demonstrated that CCTE, prepared by purifying the crude extract of *C. tinctoria* with AB‐8 macroporous resin (eluted with 50% ethanol), possessed definite antidepressant activity (Ma et al. 2022). Quantitative analysis indicated that the total polyphenol content in this fraction reached 49.7%, suggesting that these compounds may constitute the material basis for the antidepressant effects of *C. tinctoria* (Han et al. 2023). Additionally, pharmacokinetic studies conducted by our group confirmed that all 11 polyphenols were detectable in rat plasma following oral administration of CCTE. Notably, the systemic exposure of the flavonoid aglycones (OKA, ISO, TAX) was significantly higher than that of their corresponding glycosides (MR, FMR, TDG) (Han et al. 2023). This finding provided a foundation for further investigation into the active constituents and underlying mechanisms of action.

Although preliminary studies suggest the mood‐supporting potential of CCT polyphenols, their nutritional efficacy under chronic stress conditions and the underlying nutritional mechanisms remain to be systematically clarified. The present study employed a CUMS‐induced mouse model to evaluate the mood health benefits of dietary CCTE supplementation as a functional food ingredient. The nutritional functional profile of CCTE was systematically characterized across multiple dimensions relevant to health effects, including monoamine neurotransmitter levels, HPA axis homeostasis, oxidative stress status, inflammatory cytokine levels, and hippocampal neuronal protection, with a focus on elucidating its regulatory mechanism via the NLRP3/NF‐κB signaling pathway. Meanwhile, LC–MS/MS was applied for the first time to identify blood–brain barrier–permeable bioactive constituents in brain tissue after oral CCTE intake, combined with in vitro enzymatic assays to verify their MAO‐A inhibitory activity and potential synergistic interactions. This study aims to clarify the nutritional functional mechanisms and bioactive material basis of CCTE, providing scientific experimental evidence for the development and application of *Coreopsis tinctoria* polyphenols as a functional food ingredient for mood health support.

## 2 Materials and Methods

### 2.1 Materials and Reagents

The capitula of *C. tinctoria* (CCT) were collected from the Hotan region, Xinjiang, China, and authenticated by Professor Ayiguli (Department of Pharmacognosy, Xinjiang Agricultural University; Batch No. CT‐2003‐XJ). MR, FMR, TDG, QDG, FDG, OKA, ISO, TAX, CA, TCA, and FCA were synthesized in‐house; structural identity was confirmed by UV, ^1^H NMR, ^13^C NMR, and Q‐TOF MS, and purity (≥ 98.5%) was verified by HPLC. Pargyline and fluoxetine (≥ 98.5% purity) were purchased from Aladdin Biochemical Technology Co. Ltd. (Shanghai, China). β‐Glucuronidase (Type IX‐A, G7396), sulfatase (Type IV, S1629), neutral balsam (GB590), and hematoxylin (H9627) were obtained from Sigma‐Aldrich (St. Louis, MO, USA). ELISA kits for 5‐hydroxytryptamine (5‐HT, E‐EL‐0033c), 5‐hydroxyindoleacetic acid (5‐HIAA, E‐EL‐0075c), tumor necrosis factor‐α (TNF‐α, E‐EL‐M0002), interleukin‐6 (IL‐6, E‐EL‐M0001), interleukin‐1β (IL‐1β, E‐EL‐M0003), and nuclear factor kappa‐B (NF‐κB, E‐EL‐M0836c) were also obtained from Sigma‐Aldrich. Assay kits for glutathione (GSH, E‐BC‐K030‐M), catalase (CAT, E‐BC‐K031‐M), superoxide dismutase (SOD, E‐BC‐K020‐M), malondialdehyde (MDA, E‐BC‐K025‐S), corticosterone (CORT, E‐OSEL‐M001), adrenocorticotropic hormone (ACTH, E‐EL‐M0079c), and corticotropin‐releasing hormone (CRH, E‐EL‐M0351c) were purchased from Elabscience Biotechnology Co. Ltd. (Wuhan, China). Antibodies against NOD‐like receptor protein 3 (NLRP3, DF7438), phosphorylated nuclear factor kappa‐B (p‐NF‐κB(p‐P65), AF5006), and cysteine‐dependent aspartate‐specific protease‐3 (Caspase‐3, 19677‐1‐AP) were obtained from Affinity Biosciences (Cincinnati, OH, USA). Antibodies for interleukin‐1β (IL‐1β, ab283818), cysteine‐dependent aspartate‐specific protease‐1 (Caspase‐1, ab138483), and apoptosis‐associated speck‐like protein containing a CARD (ASC, ab309497) were sourced from Abcam (Cambridge, UK).

### 2.2 Preparation of CCTE

CCTE was prepared according to previously reported methods from our group with slight modifications (Han et al. 2023, 2016). Briefly, 500 g of CCT were dried at 50°C for 8 h and then pulverized (15,000 r/min, 3 min). The powder was sieved through a 50–70 mesh sieve. The sieved powder was mixed with 75% ethanol at a solid‐to‐solvent ratio of 1:20 (g:mL), soaked at 25°C for 2 h, and subsequently extracted by ultrasonication for 40 min. The extract was filtered through gauze, and the residue was re‐extracted twice with 15 volumes of 75% ethanol each time. The combined filtrates were concentrated under reduced pressure using a rotary evaporator to obtain a crude extract. This crude extract was then loaded onto an AB‐8 macroporous resin column. The column was first washed with 30% ethanol to remove other components, followed by elution with 50% ethanol (our preliminary screening indicated that this fraction possessed the optimal antidepressant activity) (Ma et al. 2022). The target eluate was collected, concentrated, and freeze‐dried to yield CCTE as a powder, with a final yield of 28%. The total flavonoid content of CCTE was determined using the sodium nitrite‐aluminum nitrate‐sodium hydroxide colorimetric method with rutin as the standard. The result showed that the total flavonoid content in CCTE was 42.2% ± 0.33% (*n* = 3).

### 2.3 Animal Grouping and CUMS Depression Model Establishment

Clean‐grade ICR male mice (weight 20 ± 2 g, age 4–6 weeks) were obtained from the Animal Experimentation Centre of Xinjiang Medical University under license number SCXK (Xin) 2023‐0001, and the experimental protocol (No. 2021100) was approved by the Ethics Committee of the Laboratory Animal Centre of Xinjiang Agricultural University. Seventy‐two mice were randomly divided into six groups (*n* = 12): the blank control group (gavage with sterile water), the CUMS model group (gavage with sterile water), the CUMS + fluoxetine group (0.01 g/kg), the CUMS + low‐dose CCTE group (0.25 g/kg), the CUMS + medium‐dose CCTE group (0.5 g/kg), and the CUMS + high‐dose CCTE group (1 g/kg). Fluoxetine was chosen as the in vivo reference antidepressant because it represents a clinically first‐line SSRI and is the most widely validated positive control in the CUMS model, enabling direct comparison of the overall behavioral and neurochemical efficacy of CCTE against a standard‐of‐care agent. It should be noted that fluoxetine is not a pathway‐specific inhibitor for MAO‐A, NF‐κB, NLRP3 inflammasome, or oxidative stress; for the in vitro MAO‐A enzymatic assay, pargyline was used as the mechanism‐specific positive control instead. Except for the blank control group, the other groups were housed singly and subjected to a 6‐week CUMS modeling process. Each day, one of the following 13 stimuli was randomly applied: 24‐h food deprivation, 24‐h wet bedding (bedding: water = 100 g: 200 mL), 5‐min swimming in 4°C ice water, 24‐h tilted cage, 24‐h water deprivation, 5‐min noise stimulation, 1‐min tail pinch (1 cm from the tail base), 5‐min horizontal shaking of the cage, 8‐h cohabitation, 5‐min tail suspension, 12‐h/12‐h reversed light–dark cycle, 2‐h body restraint, and 5‐min 45°C heat stimulation. All mice were gavaged once daily (10 mL/kg) and underwent behavioral assessment (sucrose preference test and open field test) once a week to monitor the model construction and the effect of drug intervention.

### 2.4 Sucrose Preference Test

The sucrose preference test was performed weekly for six consecutive weeks, beginning on the first day of CUMS exposure. Twenty‐four hours before each test, mice were subjected to water deprivation while maintaining ad libitum access to standard chow. During the 2‐h test session, each cage was equipped with two pre‐weighed bottles: one containing a 1% (w/v) sucrose solution and the other containing distilled water. To eliminate spatial bias, bottle positions were systematically alternated every hour. Measure the intake volumes of sucrose solution and pure water over a 2‐h period, then compute the sucrose preference ratio as a percentage.

$$\text{Sucrose preference ratio}\;\left(\%\right)=\left[\text{Sucrose solution intake}/\left(\text{sucrose solution intake}+\text{pure water intake}\right)\right]\times 100\%.$$

### 2.5 Open Field Test

The open field apparatus comprised a black, opaque square arena (45 cm × 45 cm × 45 cm). The floor was subdivided into a 5 × 5 grid of equal squares (9 cm × 9 cm each), with the central 3 × 3 zone designated as the center area and the surrounding annular region defined as the periphery. Prior to testing, each mouse underwent a 2‐min habituation period in the apparatus under identical ambient conditions. Subsequently, spontaneous locomotor and exploratory behavior was recorded for 5 min. Quantified parameters included: (i) center crossings, (ii) time spent in the center area, and (iii) rearing events. Between subjects, the arena walls and floor were thoroughly cleaned with 75% ethanol to eliminate olfactory cues and prevent carryover effects.

### 2.6 Blood and Brain Tissue Collection and Processing

Following the final drug administration and completion of behavioral assessments, mice were fasted for 12 h with unrestricted access to water to minimize dietary confounding effects on serum biomarkers. Under mild anesthesia induced by isoflurane, blood samples were collected via the retro‐orbital venous plexus. Serum separation was achieved by allowing blood samples to clot at room temperature (22°C ± 2°C) for 90 min, followed by centrifugation at 4°C and 3500*g* for 15 min. The resulting clear serum supernatant was aliquoted and stored at −80°C until analysis of inflammatory cytokines, monoamine neurotransmitters, and their major metabolites. Immediately thereafter, mice were humanely euthanized by cervical dislocation, and whole brains were rapidly excised on ice. Brains were gently rinsed in ice‐cold physiological saline to remove residual blood without mechanical damage, and hippocampi were bilaterally dissected under stereomicroscopic guidance. One hemisphere of the hippocampus was immersion‐fixed in 4% paraformaldehyde for 24 h at 4°C, then processed for histopathological evaluation and immunohistochemistry. The contralateral hippocampal hemisphere was snap‐frozen in liquid nitrogen within 60 s post‐excision and stored at −80°C for subsequent ELISA quantification of neurotransmitter levels and oxidative stress markers.

### 2.7 Hematoxylin and Eosin (H&E) Staining

Serial coronal sections (4 μm thick) were prepared from paraffin‐embedded hippocampal tissue. Following standard dewaxing and rehydration procedures, sections were stained with hematoxylin for 5 min, rinsed thoroughly with running tap water, differentiated in 1% acid alcohol for 3 s, and counterstained with eosin for 2 min. Subsequently, sections were dehydrated through a graded ethanol series, cleared in xylene, and permanently mounted with neutral balsam. Neuronal morphology was systematically evaluated in the hippocampal CA1, CA3, and dentate gyrus (DG) subregions using bright‐field microscopy at 400× magnification.

### 2.8 Nissl Staining

Paraffin sections (4 μm thick) of fixed hippocampal tissue were dewaxed and rehydrated, stained in toluidine blue staining solution at 45°C for 30 min, rinsed with distilled water, dehydrated through a graded ethanol series, differentiated with a differentiation solution, cleared, and mounted. The morphology and distribution of Nissl bodies were observed under a microscope.

### 2.9 Enzyme‐Linked Immunosorbent Assay (ELISA)

Frozen hippocampal tissue was homogenized in cold physiological saline at a 1:9 (w/v) ratio in an ice bath. After centrifugation at 4°C and 3500 rpm for 15 min, the supernatant was collected. The hippocampal homogenate supernatant and the collected serum samples were then analyzed strictly according to the instructions of the respective ELISA kits to measure the following indicators: the levels of 5‐HT and 5‐HIAA in both hippocampal homogenate and serum; the activities of SOD and CAT, and the contents of GSH and MDA in hippocampal homogenate; and the levels of CORT, CRH, ACTH, TNF‐α, IL‐6, IL‐1β, and NF‐κB in serum.

### 2.10 Immunohistochemistry (IHC)

Consecutive coronal sections were prepared from paraffin‐embedded, formalin‐fixed hippocampal tissue, aligned to the maximal coronal plane of the hippocampus. Following dewaxing in xylene and rehydration through a graded ethanol series, antigen retrieval was performed using citrate buffer (pH 6.0) under microwave heating. Endogenous peroxidase activity was quenched with 3% hydrogen peroxide for 15 min at room temperature, followed by three 5‐min washes in phosphate‐buffered saline (PBS). Nonspecific binding was blocked with 5% bovine serum albumin (BSA) in PBS for 1 h at room temperature, after which sections were incubated overnight at 4°C with primary antibodies diluted in blocking solution. After three 5‐min PBS washes, sections were incubated with horseradish peroxidase (HRP)‐conjugated secondary antibodies for 30 min at 37°C. Immunoreactive signals were visualized using 3,3′‐diaminobenzidine (DAB) substrate, and nuclei were counterstained with hematoxylin. Sections were then dehydrated in graded ethanol, cleared in xylene, and mounted with neutral balsam. Images of the hippocampal CA1 subfield were acquired under bright‐field microscopy at 400× magnification. Positive immunostaining intensity in the CA1 region was quantified as integrated optical density (IOD) using Image‐Pro Plus software (version 6.0), with consistent threshold settings and background subtraction applied across all samples.

### 2.11 Detection of Polyphenol Components in Brain Tissue

Brain tissue homogenates were prepared on ice using acetonitrile: water (1: 1, v/v) as the homogenization medium at a tissue‐to‐medium ratio of 1:4 (g:mL). For each sample, 50 μL of homogenate was aliquoted and processed in triplicate under three conditions: (a) Prototype analysis: 10 μL of 10% formic acid and 800 μL of acetonitrile containing the internal standard (IS, kaempferol, 80 ng/mL) were added. After vortexing for 5 min and centrifugation (13,000 rpm, 4°C, 10 min), 700 μL of supernatant was collected, evaporated to dryness under vacuum at 37°C, and reconstituted in 100 μL of methanol–water (3:7, v/v). After vortexing (5 min) and centrifugation, 50 μL of the supernatant was taken for LC–MS/MS analysis (injection volume: 4 μL). (b) Hydrolysis of glucuronide conjugates: 10 μL vitamin C (100 mg/mL, as antioxidant), 4 μL β‐glucuronidase (12,500 U/mL), and 14 μL sodium acetate buffer (1 mmol/L, pH 5.0) were added. Following incubation at 37°C for 3 h with shaking, the sample was processed as in (a). (c) Hydrolysis of sulfate conjugates: 10 μL vitamin C (100 mg/mL), 2 μL sulfatase (400 U/mL), and 14 μL of the same buffer were added, incubated at 37°C for 3 h, and then processed identically to (a).

LC–MS/MS analysis was performed using a validated method developed in our laboratory (Han et al. 2023). Chromatographic separation was achieved on an Agilent Poroshell 120 SB‐C18 column (2.1 mm × 50 mm, 2.7 μm) maintained at 35°C. The mobile phase consisted of solvent A (0.1% formic acid in water) and solvent B (0.1% formic acid in acetonitrile), delivered at a flow rate of 0.4 mL/min under gradient elution (program detailed in Table 1). Mass spectrometric detection was carried out using an electrospray ionization (ESI) source in negative‐ion mode with multiple reaction monitoring (MRM). Key instrumental parameters were as follows: capillary voltage, 3.5 kV; desolvation temperature, 325°C; desolvation gas flow, 5 L/min; sheath gas temperature, 350°C; sheath gas flow, 11 L/min. Compound‐specific MRM transitions, declustering potentials, and collision energies are provided in Table 2. Quantification was performed using the internal standard method with kaempferol as the internal standard. Calibration curves were constructed over the linear dynamic range of each analyte, and concentrations in brain tissue were reported as nanograms per gram of wet weight (ng/g).

**TABLE 1:** Gradient program for the determination of 11 polyphenols.

Time (min)	*A* (%)	*B* (%)
Initial	89	11
1.34	88	12
2.67	87	13
5.33	84	16
6.67	75	25
8.9	75	25
9.0	50	50
11	50	50
11.1	89	11
13.5	89	11

**TABLE 2:** The precursor ions, product ions, fragmentor and collision energy for each analyte and kaempferol (internal standard, IS) in the LC–MS/MS analysis.

Analyte	Precursor ions (m/z)	Product ions (m/z)	Fragmentor (V)	Collision energy (V)
MR	449.1	287.0	150	17
FMR	449.1	287.0	160	17
TDG	465.1	303.0	160	10
QDG	479.1	317.0	175	24
FDG	449.1	287.0	165	14
OKA	287.0	135.0	110	21
ISO	287.0	135.0	120	19
TAX	303.0	125.0	110	18
CA	353.0	191.0	80	11
TCA	515.1	353.0	130	13
FCA	515.1	353.1	130	16
IS	285.0	285.0	165	20

### 2.12 MAO‐A Activity Assay

Monoamine oxidase (MAO) was isolated from CUMS mouse brain tissue. Brain tissue was homogenized in pre‐cooled 0.3 mol/L sucrose solution at a ratio of 1:35 (g/mL) on ice. The homogenate was centrifuged at 4°C and 1500 rpm for 10 min, and the supernatant was collected. This supernatant was further centrifuged at 4°C and 10,000 rpm for 30 min to obtain a pellet. The pellet was resuspended in 0.3 mol/L sucrose solution (0.8 mL per gram of brain tissue), followed by the addition of 1.2 mol/L sucrose solution (8 mL per gram of brain tissue) and gentle mixing. The suspension was then centrifuged at 4°C and 10,500 rpm for 40 min. The resulting pellet was washed once by resuspension in 50 mmol/L phosphate buffer (pH 7.4) and centrifugation under identical conditions. Finally, the pellet was resuspended in phosphate buffer (0.4 mL per gram of tissue) to obtain the MAO enzyme source. The protein concentration was determined using the BCA method and adjusted to 1.5 mg/mL for subsequent use.

Enzyme activity assays were conducted in 96‐well microplates. Each well received 20 μL of MAO solution (1.5 mg/mL) and 50 μL of 50 mmol/L phosphate buffer (pH 7.0). After gentle mixing, plates were pre‐incubated at 37°C for 10 min. Subsequently, 5 μL of 5‐HT (2 mmol/L, final concentration 100 μmol/L) and 25 μL of test compound solution (pargyline, OKA, ISO, TAX, or their equimolar 1:1:1 mixture) were added to achieve a total reaction volume of 100 μL. Six serial dilutions (0.1, 1.0, 10, 20, 50, and 100 μmol/L) were tested for each compound. The final methanol concentration in the reaction system was maintained below 1%. Control groups included: (i) negative control (buffer in place of test compound), (ii) blank control (no 5‐HT substrate; otherwise identical to test group), (iii) positive control (100 μmol/L pargyline), and (iv) enzyme‐inactivated control (MAO pre‐heated at 90°C for 30 min, then cooled on ice before use). Reactions proceeded at 37°C for 60 min. Five minutes prior to termination, 10 μL of 1 mol/L HCl was added to stabilize the product. The reaction was quenched by adding 200 μL of ice‐cold acetonitrile, followed by vortexing for 5 min and centrifugation at 4°C and 13,000*g* for 10 min. A 10 μL aliquot of the clear supernatant was injected onto the HPLC system. Chromatographic separation was achieved on an Agilent SB‐aq column (150 mm × 4.6 mm, 5 μm) maintained at 30°C. The mobile phase consisted of solvent A (ultrapure water) and solvent B (acetonitrile) delivered at 0.7 mL/min under the following linear gradient: 0–3.0 min, 10% B; 3.0–3.1 min, 10%–50% B; 3.1–5.0 min, 50% B; 5.0–5.1 min, 50%–10% B; 5.1–8.0 min, 10% B. Detection was performed at 254 nm, with an injection volume of 10 μL. Under these conditions, 5‐hydroxyindoleacetic acid (5‐HIAA), the oxidative deamination product of 5‐HT, eluted at approximately 6.34 min.

MAO‐A inhibitory activity was calculated as percentage inhibition using the formula: Inhibition (%) = [(*A* − *A*_0_) − (*A*_*i*_ − *A*_*i*0_)] / (*A* − *A*_0_) × 100. A represents the product peak area in the negative control, *A*_0_ is the peak area in the enzyme‐inactivated control, *A*_*i*_ is the peak area in each treatment group, and *A*_*i*0_ is the peak area in the corresponding blank control for that treatment. Dose–response curves were generated by plotting MAO‐A percentage inhibition against the logarithmic concentration of each test compound. Nonlinear regression with a four‐parameter logistic (4PL) model (log[inhibitor] vs. response—variable slope, Hill slope model) was applied to fit the sigmoidal dose–response curves using GraphPad Prism 8.0 software (GraphPad Software, San Diego, CA, USA). The half‐maximal inhibitory concentration (IC_50_) value for each compound was computed directly from the fitted curve. Three technical replicates were performed for each concentration point to ensure the reliability of curve fitting.

### 2.13 Statistical Analysis

All statistical analyses and graphical representations were performed using GraphPad Prism 8.0. Data were expressed as mean ± standard error of the mean (SEM). For comparisons across the multiple experimental groups, one‐way analysis of variance (one‐way ANOVA) was first conducted to detect overall intergroup differences, followed by Tukey's post hoc test for pairwise multiple comparisons. Welch's correction was applied when group variances were unequal. Statistical significance was defined as *p* < 0.05.

## 3 Results

### 3.1 CCTE Improves Behavior in CUMS‐Induced Depressed Mice

Throughout the modeling period, mice in the blank control group maintained normal physiological and behavioral status, whereas mice in the CUMS model group gradually displayed depressive‐like behaviors, including ruffled fur, reduced food and water intake, slowed weight gain, huddling, and sluggish responses; these deficits were markedly attenuated by CCTE or fluoxetine. One week after modeling, the weight gain rate in the CUMS group was significantly lower than that in the control group (−6.4% ± 1.0% vs. 19.1% ± 2.0%; *p* < 0.001) (Figure 1A), and it remained suppressed through week 6 (4.7% ± 2.4% vs. 55.6% ± 3.3%; *p* < 0.001). During weeks 4–6, CCTE and fluoxetine significantly increased the weight gain rate in a dose‐dependent manner; by week 6, the low‐, medium‐, and high‐dose CCTE groups and the fluoxetine group reached 18.7% ± 1.5%, 30.7% ± 2.8%, 38.8% ± 5.9%, and 28.3% ± 3.5%, respectively (all *p* < 0.001 vs. CUMS). No deaths occurred during the experiment.

**FIGURE 1:**
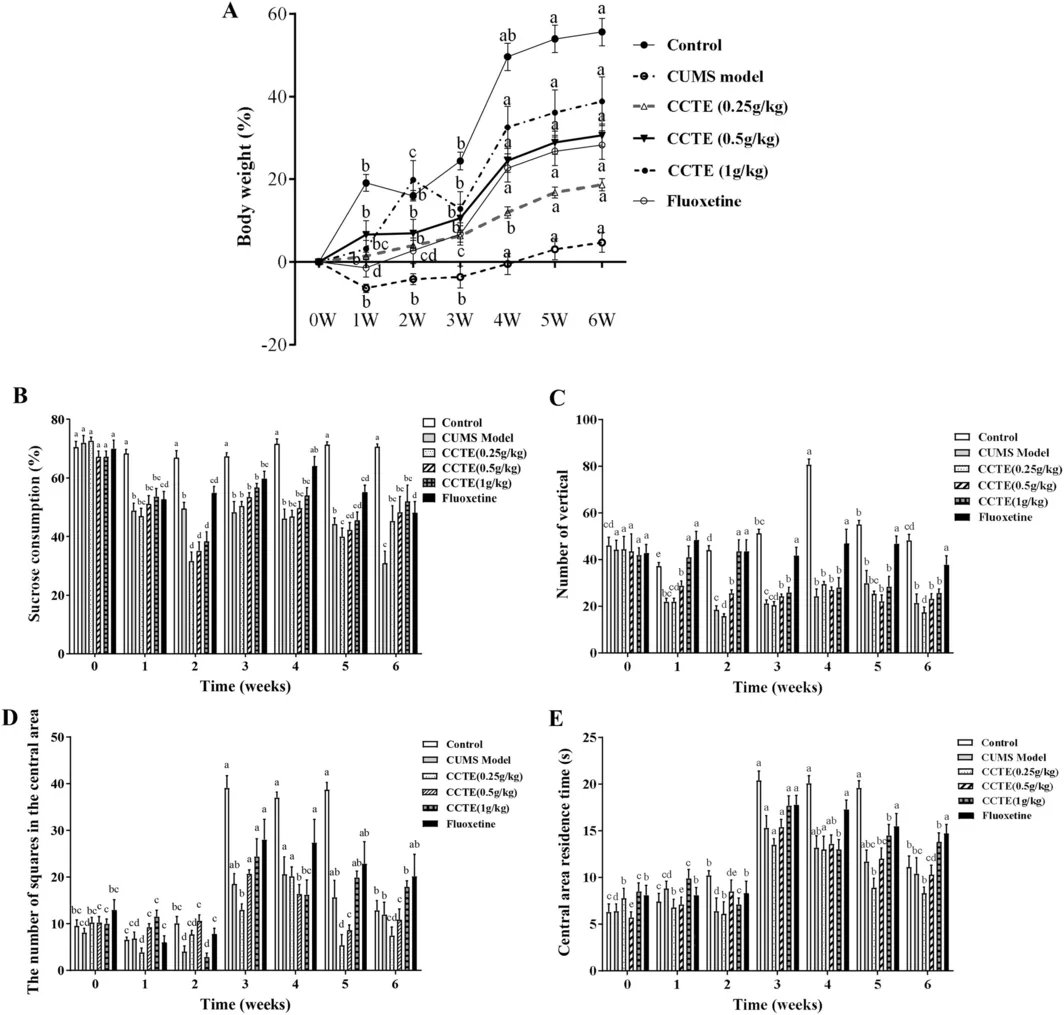
Effects of CCTE on behavioral performance in CUMS‐induced depressive mice. (A) Percentage change in body weight of mice. (B) Sucrose preference rate; (C) Rearing counts; (D) Number of central area crossings; (E) Time spent in the central area. Different letters indicate statistically significant differences (*p* < 0.05) by one‐way ANOVA followed by Tukey's post hoc test.

Sucrose preference was comparable among groups at baseline (~70%), but was markedly reduced in the CUMS model group, confirming anhedonia (30.9% ± 4.1% vs. 70.6% ± 1.0% in controls at week 6; *p* < 0.001) (Figure 1B). Fluoxetine significantly elevated sucrose preference from week 2 onward, whereas all CCTE doses did so from week 3 onward (*p* < 0.05); at week 6, preference in the low‐, medium‐, and high‐dose CCTE and fluoxetine groups was 45.3% ± 5.3%, 48.3% ± 5.4%, 52.0% ± 5.5%, and 48.1% ± 3.8%, respectively (all *p* < 0.01 vs. CUMS), indicating dose‐dependent, delayed‐onset restoration. In the open field test (Figure 1C–E), CUMS significantly reduced rearing counts during weeks 1–3 (e.g., 18.4 ± 1.7 vs. 44.0 ± 1.9 at week 2; *p* < 0.01), and fluoxetine and high‐dose CCTE significantly restored this parameter (43.6 ± 4.8 and 42.5 ± 4.7 at week 2; both *p* < 0.01). Similarly, central area crossings were significantly fewer in CUMS mice during weeks 3–5 (e.g., 18.5 ± 2.2 vs. 39.1 ± 2.6 at week 3; *p* < 0.01), and fluoxetine and high‐dose CCTE significantly increased crossings (28.0 ± 4.4 and 24.4 ± 3.9 at week 3; both *p* < 0.01); at week 6, although CUMS no longer differed from controls (12.0 ± 2.6 vs. 12.9 ± 2.0), both treatments remained significantly higher than the model group (20.2 ± 4.7 and 17.9 ± 1.3; both *p* < 0.01). Time spent in the central area was significantly shorter in CUMS mice from week 2 onward (e.g., 15.3 ± 1.3 s vs. 20.4 ± 1.0 s at week 3; *p* < 0.01), and was significantly prolonged by fluoxetine and high‐dose CCTE (17.8 ± 1.0 s and 17.7 ± 1.0 s at week 3; both *p* < 0.01). Collectively, high‐dose CCTE effectively reversed CUMS‐induced exploratory deficits and anxiety‐like behaviors with efficacy comparable to fluoxetine, whereas low‐ and medium‐dose CCTE showed no significant ameliorative effects, indicating a dose‐dependent pattern of action.

### 3.2 CCTE Suppressed Serum Inflammatory Cytokine Levels

As depicted in Figure 2, serum concentrations of all measured inflammatory mediators were significantly elevated in the CUMS model group relative to the blank control group, indicating that CUMS induced a systemic inflammatory response and activated the NF‐κB signaling pathway, with both fluoxetine and CCTE reducing these factors to varying extents. Quantitatively, compared with the blank control group, CUMS increased serum IL‐1β, IL‐6, TNF‐α, and NF‐κB by approximately 32.78%, 45.06%, 52.55%, and 48.52%, respectively; high‐dose CCTE (1 g/kg) reduced these four inflammatory mediators by approximately 51.01%, 35.08%, 51.31%, and 47.40% versus the CUMS group (all *p* < 0.01), restoring them to levels comparable to those of the blank control group and to fluoxetine. Specifically, serum IL‐1β was significantly increased in the CUMS model group (*p* < 0.05) (Figure 2A): low‐dose CCTE produced no significant change (*p* > 0.05), whereas medium‐dose CCTE, high‐dose CCTE, and fluoxetine all significantly reduced IL‐1β levels (*p* < 0.01, *p* < 0.001, and *p* < 0.001, respectively). A similar pattern was observed for IL‐6 (Figure 2B), which was significantly elevated in the CUMS group (*p* < 0.01); low‐dose and medium‐dose CCTE showed downward trends without statistical significance (*p* > 0.05), while high‐dose CCTE and fluoxetine both significantly suppressed IL‐6 (*p* < 0.01 and *p* < 0.001, respectively). Serum TNF‐α was markedly higher in the CUMS model group (*p* < 0.01) (Figure 2C): low‐dose CCTE had no significant effect (*p* > 0.05), whereas medium‐dose CCTE, high‐dose CCTE, and fluoxetine all significantly reduced TNF‐α concentrations (*p* < 0.01, *p* < 0.001, and *p* < 0.001, respectively). Regarding NF‐κB (Figure 2D), the CUMS‐induced increase was significant (*p* < 0.001); low‐ and medium‐dose CCTE produced decreasing trends that did not reach statistical significance (*p* > 0.05), while high‐dose CCTE and fluoxetine both elicited robust, statistically significant reductions (*p* < 0.001 and *p* < 0.001, respectively). In summary, low‐dose CCTE failed to significantly affect any of the four biomarkers; medium‐dose CCTE significantly decreased TNF‐α and IL‐1β, with only non‐significant downward trends for IL‐6 and NF‐κB; and notably, both fluoxetine and high‐dose CCTE exerted robust and statistically significant suppression of all four inflammatory mediators.

**FIGURE 2:**
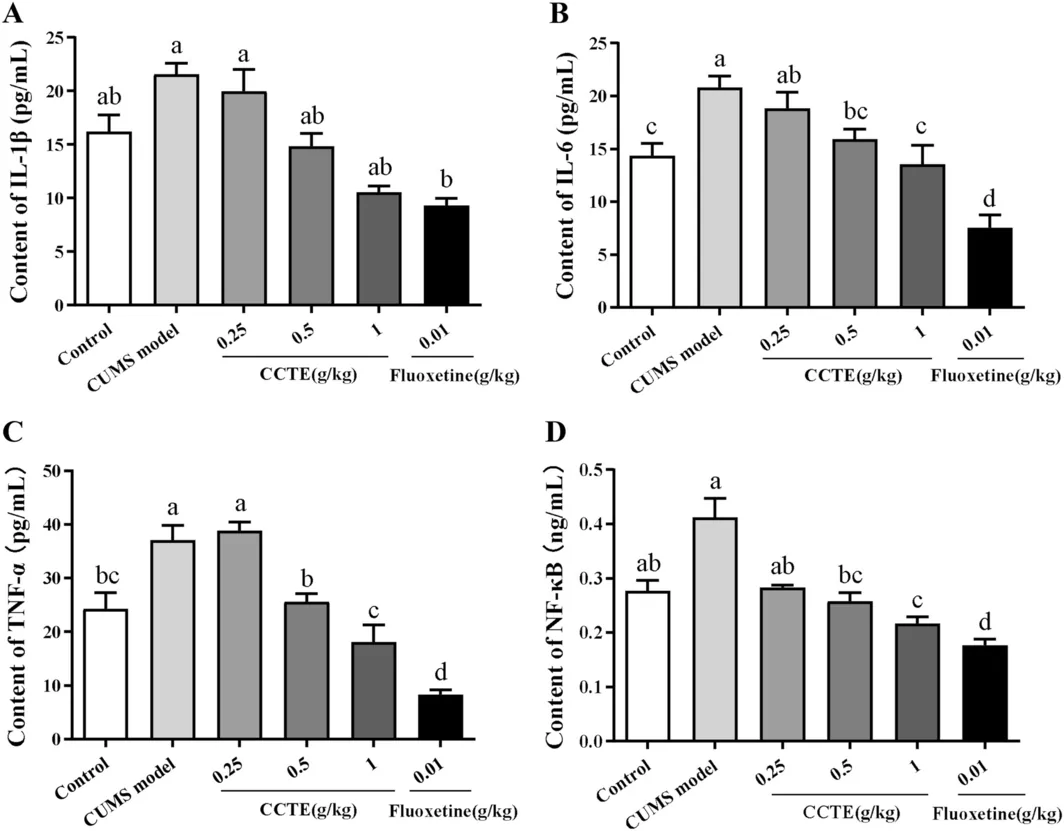
Effects of CCTE on inflammatory cytokine levels in CUMS mice serum. (A) IL‐1β; (B) IL‐6; (C) TNF‐α; (D) NF‐κB. Different letters indicate statistically significant differences (*p* < 0.05) by one‐way ANOVA followed by Tukey's post hoc test.

### 3.3 CCTE Regulates 5‐HT and 5‐HIAA Levels in Serum and Brain Tissue

Figure 3 illustrates the concentrations of 5‐HT and 5‐HIAA in the serum and brain tissue of each experimental group. Compared with the blank control group, the serum 5‐HT concentration in the CUMS model group was significantly decreased (*p* < 0.001), with a reduction of approximately 36.7%, while the serum 5‐HIAA concentration was significantly increased (*p* < 0.001), with an increase of approximately 44.8%. In brain tissue, the 5‐HT level in the CUMS model group also decreased significantly (*p* < 0.05), with a reduction of approximately 27.6%, whereas the 5‐HIAA level increased significantly (*p* < 0.05), with an increase of approximately 24.6%. These results collectively indicate that CUMS induced disturbances in both central and peripheral monoaminergic metabolism.

**FIGURE 3:**
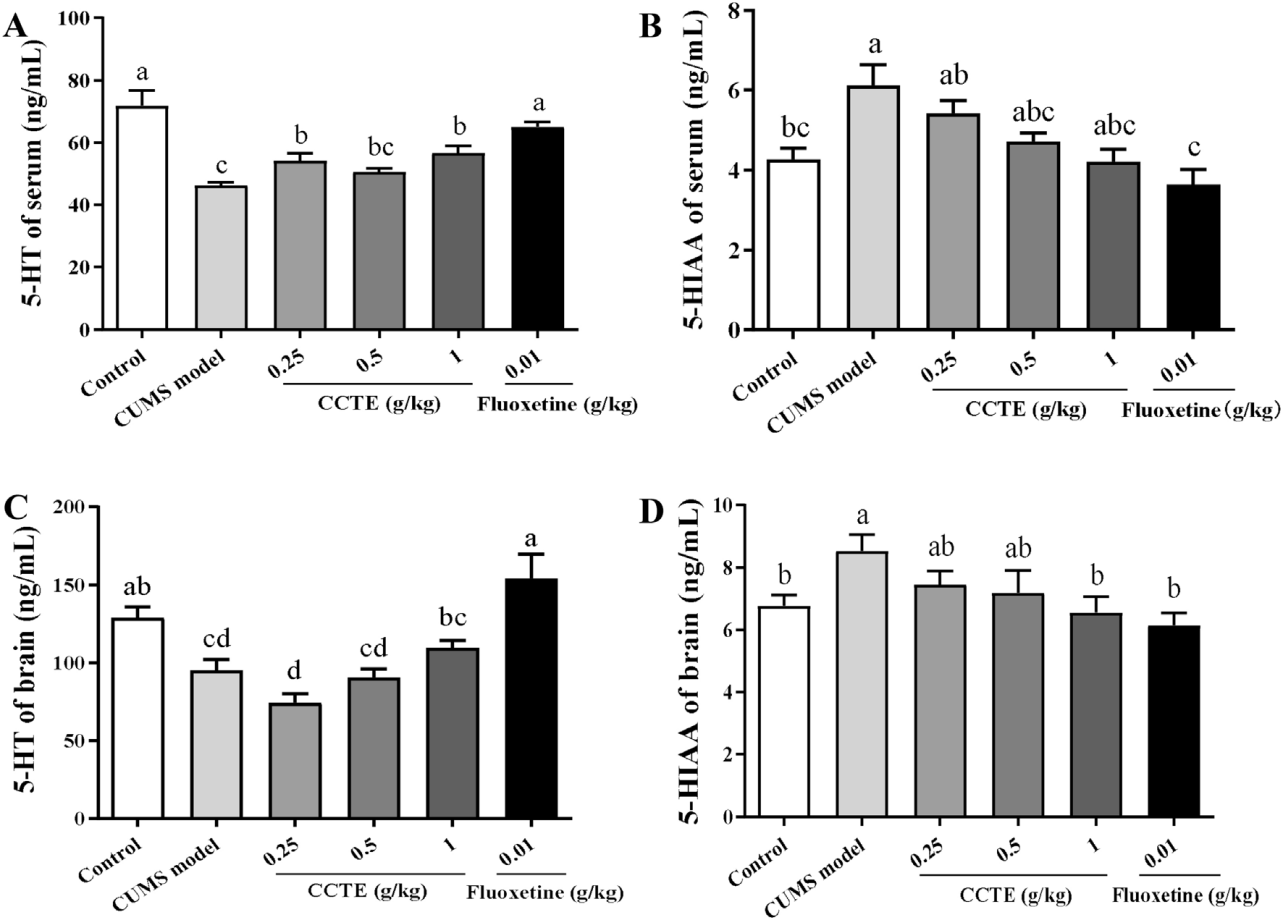
Effects of CCTE on 5‐HT and 5‐HIAA levels in serum and brain tissue of CUMS mice. (A) 5‐HT levels in serum; (B) 5‐HIAA levels in serum; (C) 5‐HT levels in brain tissue; (D) 5‐HIAA levels in brain tissue. Different letters indicate statistically significant differences (*p* < 0.05) by one‐way ANOVA followed by Tukey's post hoc test.

After administration of different doses of CCTE, the monoaminergic indices in mice exhibited a general trend toward recovery, with the high‐dose group showing the most pronounced effect. In serum, low‐, medium‐, and high‐dose CCTE increased 5‐HT by approximately 17.3% (*p* < 0.05), 9.7% (*p* < 0.05), and 23.6% (*p* < 0.05), respectively, compared with the CUMS model group, and decreased 5‐HIAA by approximately 11.3% (*p* < 0.05), 23.5% (*p* < 0.01), and 19.4% (*p* < 0.01), respectively. In brain tissue, all doses showed similar ameliorative effects. Notably, high‐dose CCTE reduced the decrease in brain 5‐HT from 27.6% in the CUMS model group to approximately 15.6% (*p* < 0.05) and lowered the increase in brain 5‐HIAA from 24.6% to approximately 10.5% (*p* < 0.01), demonstrating the most pronounced recovery. Taken together, CCTE concentration‐dependently increased 5‐HT bioavailability and inhibited its excessive metabolism, effectively correcting the CUMS‐induced monoaminergic imbalance. The efficacy of high‐dose CCTE approached that of the positive control fluoxetine, further validating the potential of CCTE in modulating the monoaminergic system.

### 3.4 CCTE Regulates Neuroendocrine Homeostasis

As depicted in Figure 4, serum concentrations of CRH, ACTH, and CORT were all significantly elevated in the CUMS model group relative to the blank control group—by approximately 94.57% (*p* < 0.001), 57.75% (*p* < 0.001), and 18.93% (*p* < 0.01), respectively—confirming successful establishment of a hyperactive HPA axis and associated neuroendocrine dysregulation. CCTE reversed these elevations in a clear dose‐dependent manner. Low‐dose CCTE had no significant effect on CRH or ACTH (*p* > 0.05). Medium‐dose CCTE significantly reduced CRH and CORT (both *p* < 0.01) but did not significantly alter ACTH (*p* > 0.05). High‐dose CCTE significantly suppressed all three hormones, reducing CRH, ACTH, and CORT by approximately 19.96%, 18.76%, and 23.62%, respectively, versus the CUMS group (*p* < 0.01 to *p* < 0.001). The magnitude of these reductions was comparable to that achieved by fluoxetine, which likewise significantly suppressed all three hormones (CRH and CORT: *p* < 0.001 for both groups; ACTH: *p* < 0.01 for high‐dose CCTE and *p* < 0.001 for fluoxetine), with no significant difference between the two groups for any parameter (*p* > 0.05). Collectively, these findings demonstrate that CCTE ameliorates CUMS‐induced neuroendocrine dysfunction via dose‐dependent normalization of HPA axis activity.

**FIGURE 4:**
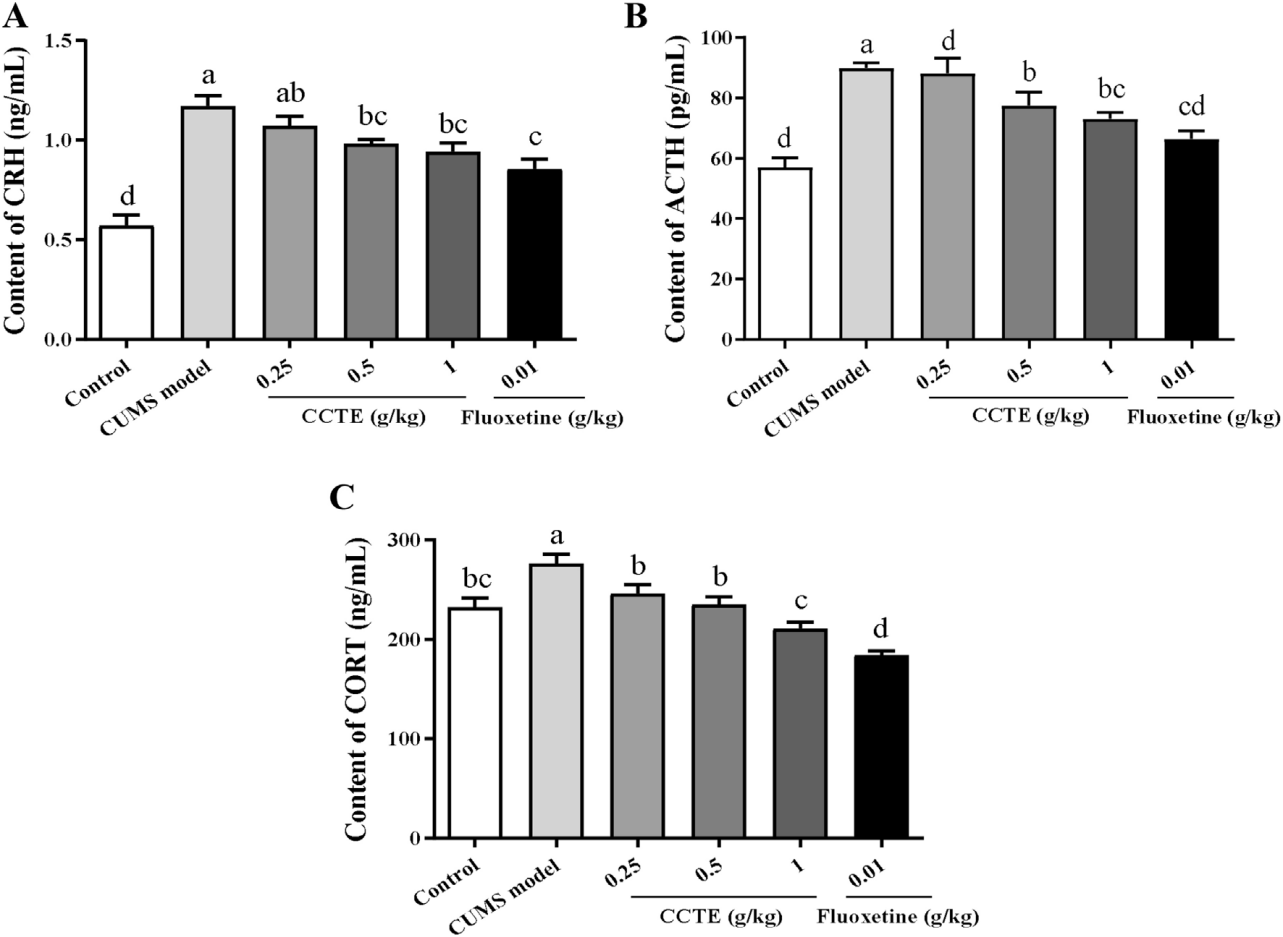
Effects of CCTE on serum CORT, ACTH, and CRH levels in CUMS mice. (A) CRH; (B) ACTH; (C) CORT. Different letters indicate statistically significant differences (*p* < 0.05) by one‐way ANOVA followed by Tukey's post hoc test.

### 3.5 CCTE Ameliorated Oxidative Stress in Brain Tissue

As shown in Figure 5, the CUMS model group exhibited significant disruption of redox homeostasis in brain tissue, evidenced by increased lipid peroxidation and compromised endogenous antioxidant defense. Compared with the blank control group, SOD activity was significantly decreased by 41.47% (Figure 5A; *p* < 0.05), MDA content was markedly elevated by 72.35% (Figure 5B; *p* < 0.001), CAT activity increased compensatorily by 114.17% (Figure 5C; *p* < 0.001), and GSH content rose by 50.83% (Figure 5D; *p* < 0.05). CCTE reversed these abnormalities in a dose‐dependent manner. Medium‐dose CCTE significantly increased SOD activity (*p* < 0.05) and reduced MDA levels (*p* < 0.01) and CAT activity (*p* < 0.05), but did not significantly restore GSH (*p* > 0.05). High‐dose CCTE (1 g/kg) and fluoxetine both significantly ameliorated all four indices: SOD activity was increased (both *p* < 0.01), MDA levels were reduced (*p* < 0.001 and *p* < 0.05, respectively), CAT activity was normalized (both *p* < 0.01), and GSH content was significantly restored (both *p* < 0.05); high‐dose CCTE increased SOD activity by approximately 80.21% and decreased GSH, MDA, and CAT activities by approximately 29.41%, 34.09%, and 21.27%, respectively, relative to the CUMS group (*p* < 0.05 to *p* < 0.001), with improvements comparable in magnitude to those induced by fluoxetine across all four biomarkers. Collectively, these results demonstrate that CCTE alleviates CUMS‐induced oxidative damage in brain tissue in a dose‐dependent manner, primarily by enhancing enzymatic antioxidant defenses (SOD), restoring non‐enzymatic antioxidant reserves (GSH), and attenuating lipid peroxidation (MDA), thereby promoting the re‐establishment of cerebral redox balance.

**FIGURE 5:**
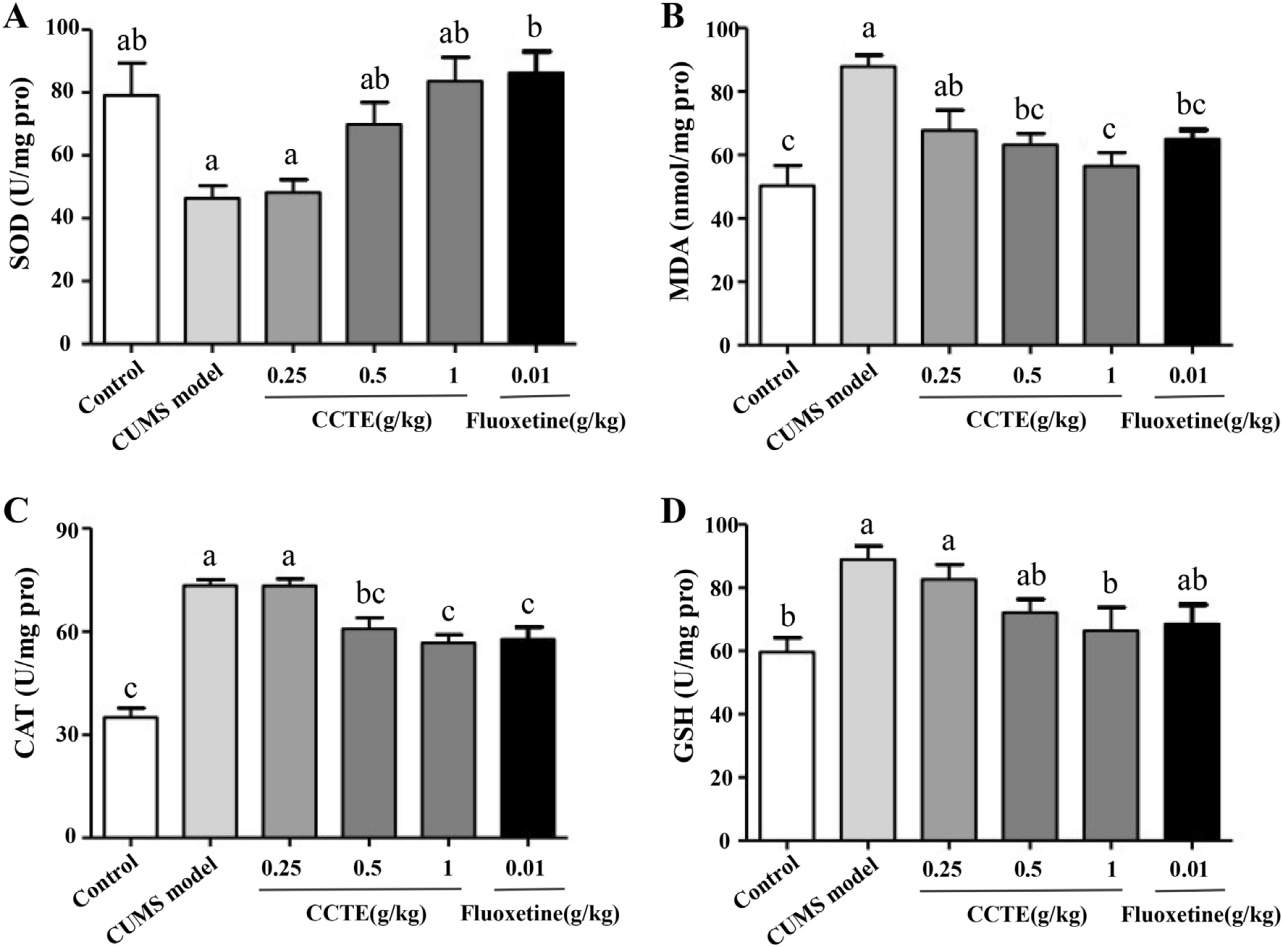
Effects of CCTE on oxidative stress in brain tissue of CUMS mice. (A) SOD; (B) MDA; (C) CAT; (D) GSH. Different letters indicate statistically significant differences (*p* < 0.05) by one‐way ANOVA followed by Tukey's post hoc test.

### 3.6 CCTE Ameliorated Hippocampal Pathomorphology in CUMS Mice

The hippocampus comprised three principal subregions: the cornu ammonis 1 (CA1) and cornu ammonis 3 (CA3) areas, and the dentate gyrus (DG) (Figure S1). Hematoxylin and eosin (H&E) staining (Figure 6A) revealed that, at 400× magnification, neurons in all three subregions of the blank control group displayed preserved cellular architecture—characterized by tightly packed somata, distinct nucleoli, abundant and homogeneous cytoplasm, and an absence of nuclear condensation or fragmentation. In contrast, the CUMS model group exhibited pronounced histopathological alterations: neuronal disarray, somatic shrinkage, cytoplasmic rarefaction, nuclear pyknosis, and focal neuronal loss in CA1, CA3, and DG. Quantitative analysis confirmed that the number of intact neurons in the CA1, CA3, and DG regions was significantly decreased in the CUMS model group relative to the blank control group (all *p* < 0.01). Quantitatively, the numbers of intact neurons in the CA1, CA3, and DG subregions were reduced by approximately 47.46%, 61.42%, and 67.12%, respectively, in the CUMS model group relative to the blank control group. Fluoxetine treatment markedly attenuated these changes, with neurons regaining near‐normal density, cytoplasmic basophilia, and nuclear integrity. Notably, CCTE administration produced dose‐dependent neuroprotective effects: low, medium and high‐dose groups showed progressive restoration of neuronal packing density, cytoplasmic volume, and nuclear morphology. Quantitatively, high‐dose CCTE significantly increased the number of intact neurons in all three hippocampal subregions (CA1, CA3, DG; all *p* < 0.01 vs. CUMS model group). The corresponding increases in intact‐neuron numbers in CA1, CA3, and DG were approximately 77.84%, 137.62%, and 172.26%, respectively, versus the CUMS group, restoring the counts to approximately 93.42%, 91.53%, and 90.81% of the blank‐control levels. These findings suggested that the CUMS‐induced depressive state could lead to structural degeneration of hippocampal neurons, and that both fluoxetine and CCTE could exert potent dose‐responsive protective effects on hippocampal neuropathology under this condition.

**FIGURE 6:**
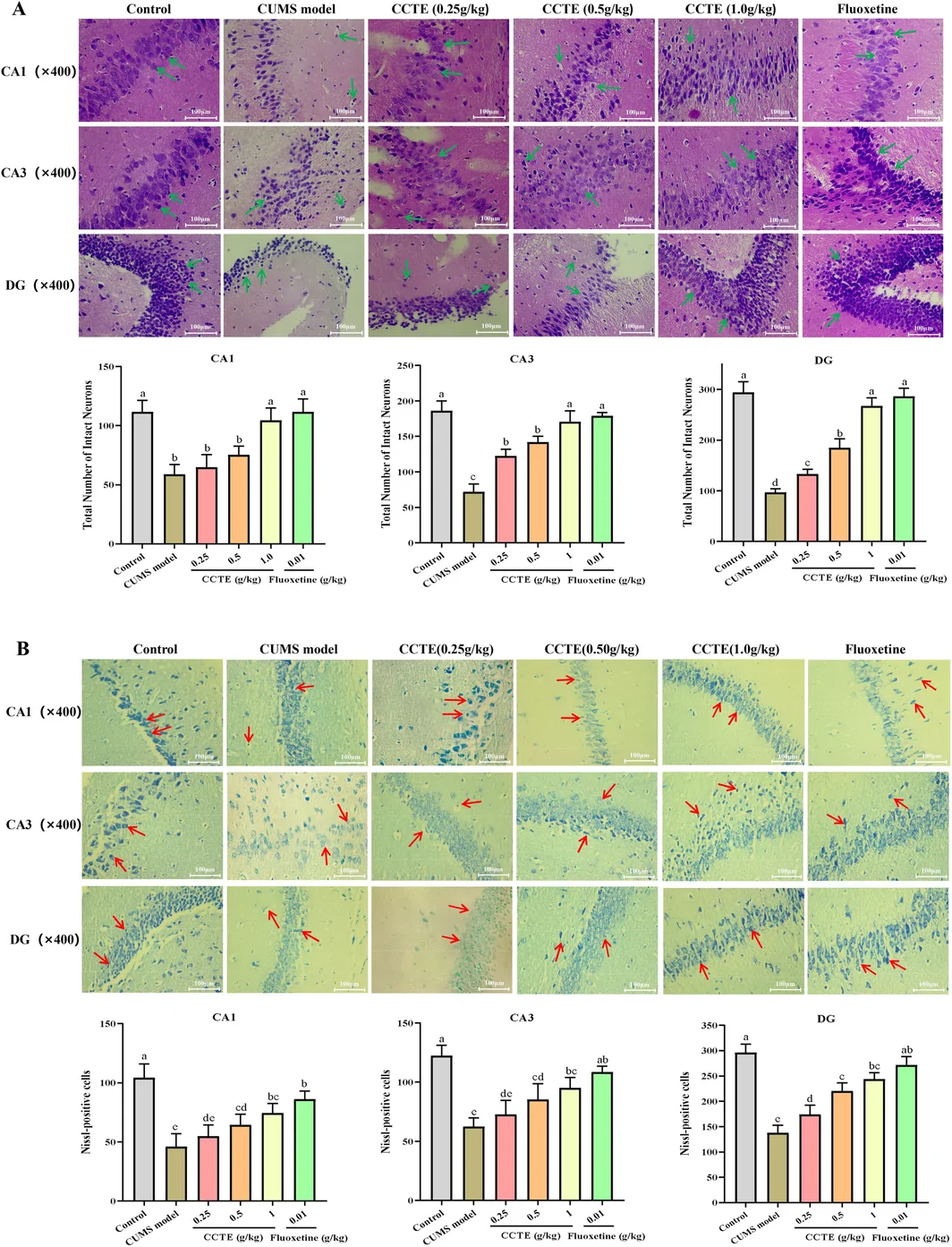
Effects of CCTE on hippocampal histopathological morphology in CUMS‐depressed mice. (A) Representative H&E‐stained sections of the hippocampus (×400) and quantitative analysis of intact neuron numbers in the CA1, CA3, and DG subregions. (B) Representative Nissl‐stained sections of the hippocampus (×400) and quantitative analysis of Nissl‐positive neuron counts in the CA1, CA3, and DG subregions. Different letters indicate statistically significant differences (*p* < 0.05) by one‐way ANOVA followed by Tukey's post hoc test.

Nissl bodies served as reliable histological markers for evaluating the severity of neuronal damage, with both their density and staining intensity exhibiting a positive correlation with the extent of depressive‐like behaviors. Nissl staining revealed that neurons in the hippocampal CA1, CA3, and DG subregions of the blank control group displayed orderly alignment, compact cytoarchitecture, and abundant, uniformly distributed Nissl bodies with sharp morphological definition (Figure 6B). In contrast, the CUMS model group exhibited marked neuronal disorganization—characterized by loosely arranged cells, diminished Nissl body staining intensity, and partial dissolution of Nissl substance. Quantitative counting demonstrated a statistically significant reduction in Nissl‐positive neuron counts across all three subregions in the CUMS model group (all *p* < 0.001 vs. blank control). Quantitatively, Nissl‐positive neuron counts in CA1, CA3, and DG were reduced by approximately 55.91%, 49.04%, and 53.45%, respectively, in the CUMS group. Fluoxetine treatment partially restored neuronal architecture: hippocampal subregions showed improved cellular alignment, enhanced Nissl staining clarity, and significantly increased Nissl‐positive neuron numbers compared with the CUMS model group (all *p* < 0.01). Notably, CCTE administration produced dose‐dependent neuroprotective effects: with escalating oral doses, neuronal packing density and spatial regularity increased progressively in CA1, CA3, and DG; Nissl staining intensity intensified; and the number of Nissl‐positive neurons rose significantly in a dose‐responsive manner. High‐dose CCTE significantly elevated Nissl‐positive neuron counts in CA1, CA3, and DG (all *p* < 0.01 vs. CUMS model group). The corresponding increases in Nissl‐positive neuron counts in the three subregions were approximately 61.57%, 52.38%, and 76.69%, respectively, versus the CUMS group, approaching the counts in the fluoxetine and blank control groups. Collectively, these findings demonstrated that chronic unpredictable mild stress induced structural damage to hippocampal neurons—including Nissl body depletion—in mice, and that both CCTE and fluoxetine effectively mitigated CUMS‐induced hippocampal neuronal pathology.

### 3.7 CCTE Inhibited Hippocampal NLRP3 Inflammasome Activation

Immunohistochemical analysis of the hippocampal CA1 region revealed distinct expression patterns of inflammasome‐related proteins, as illustrated in Figure 7. Caspase‐1 immunoreactivity (Figure 7A) was minimal in control sections, whereas the CUMS group exhibited markedly increased cytoplasmic staining with an intense brownish‐yellow signal. This CUMS‐induced upregulation was not significantly altered by low‐ or medium‐dose CCTE but was substantially reduced by high‐dose CCTE. A similar trend was observed for ASC (Figure 7B): staining was faint in controls and prominently elevated following CUMS. Low‐dose CCTE produced only a modest reduction, whereas medium‐dose CCTE, high‐dose CCTE, and fluoxetine each significantly attenuated ASC immunoreactivity. IL‐1β expression (Figure 7C) was strongly induced in the CUMS group, and this induction was significantly suppressed by medium‐dose CCTE as well as by high‐dose CCTE and fluoxetine. NLRP3 expression was dramatically upregulated in CUMS animals and dose‐dependently suppressed by CCTE treatment, with high‐dose CCTE and fluoxetine achieving significant reductions (Figure 7D). Quantitatively, relative to the blank control group, CUMS increased the relative integrated optical density (IOD) of caspase‐1, ASC, IL‐1β, and NLRP3 immunostaining by approximately 119.52%, 195.46%, 129.31%, and 148.93%, respectively (all *p* < 0.01); high‐dose CCTE (1 g/kg) reduced these IOD values by approximately 31.37%, 32.69%, 32.13%, and 56.82% versus the CUMS group (*p* < 0.01 to *p* < 0.001), with an overall inhibitory extent comparable to that of fluoxetine. Collectively, these findings demonstrate that CUMS may induce canonical NLRP3 inflammasome activation in hippocampal neurons, involving transcriptional priming and assembly that lead to caspase‐1‐mediated IL‐1β release. CCTE exerted a dose‐dependent inhibitory effect on all four components, and high‐dose CCTE suppressed the immunoreactivity of NLRP3, ASC, caspase‐1, and IL‐1β to an extent comparable to fluoxetine, thereby effectively inhibiting central pro‐inflammatory signaling.

**FIGURE 7:**
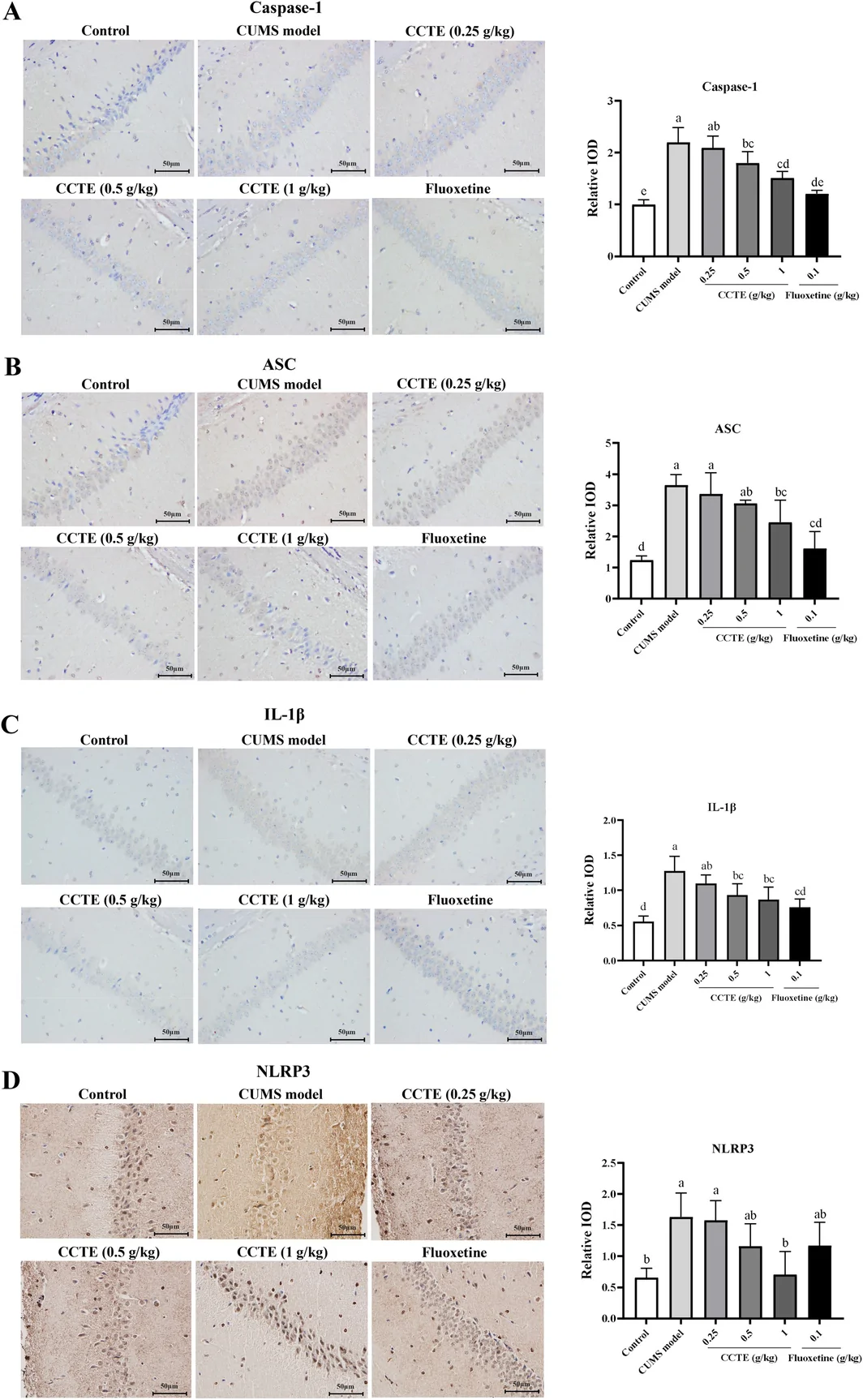
Effects of CCTE on NLRP3‐related protein expression in the hippocampal region of CUMS mice. (A) Caspase‐1; (B) ASC; (C) IL‐1β; (D) NLRP3. Different letters indicate statistically significant differences (*p* < 0.05) by one‐way ANOVA followed by Tukey's post hoc test.

### 3.8 CCTE Attenuated NF‐κB Signaling Pathway

Figure 8 illustrates that, compared with the blank control group, the CUMS model group exhibited a highly significant increase in immunoreactivity for p‐NF‐κB(p‐P65), p‐IκBα and cleaved caspase‐3 in the hippocampal CA1 region. Quantitatively, relative to the blank control group, CUMS increased the relative IOD of cleaved caspase‐3, p‐IκBα, and p‐NF‐κB (p‐p65) by approximately 315.28%, 74.54% and 435.27%, respectively (all *p* < 0.001); CCTE at 1 g/kg reduced the levels of these three proteins by approximately 56.12%, 32.08% and 55.08% (all *p* < 0.01), and the reductions induced by high‐dose CCTE were comparable in magnitude to those induced by fluoxetine. This pattern is consistent with CUMS‐induced IκBα phosphorylation and subsequent proteasomal degradation, leading to nuclear translocation and transcriptional activation of NF‐κB, and concurrent activation of the mitochondrial apoptotic pathway in hippocampal neurons. Treatment with fluoxetine or CCTE (0.5–1 g/kg) significantly attenuated immunoreactivity for p‐NF‐κB and p‐IκBα, as well as cleaved caspase‐3 expression (*p* < 0.01). These findings indicate that CCTE suppresses the canonical NF‐κB signaling cascade, thereby reducing transcription of downstream pro‐inflammatory genes and mitigating neuronal apoptosis. Importantly, the parallel inhibition of both NLRP3 inflammasome components and upstream NF‐κB signaling molecules supports a coordinated mechanism: by suppressing NF‐κB–dependent priming (e.g., transcriptional upregulation of NLRP3 and pro‐IL‐1β), CCTE limits inflammasome assembly and interrupts the reciprocal amplification between NF‐κB activation and NLRP3‐driven inflammation.

**FIGURE 8:**
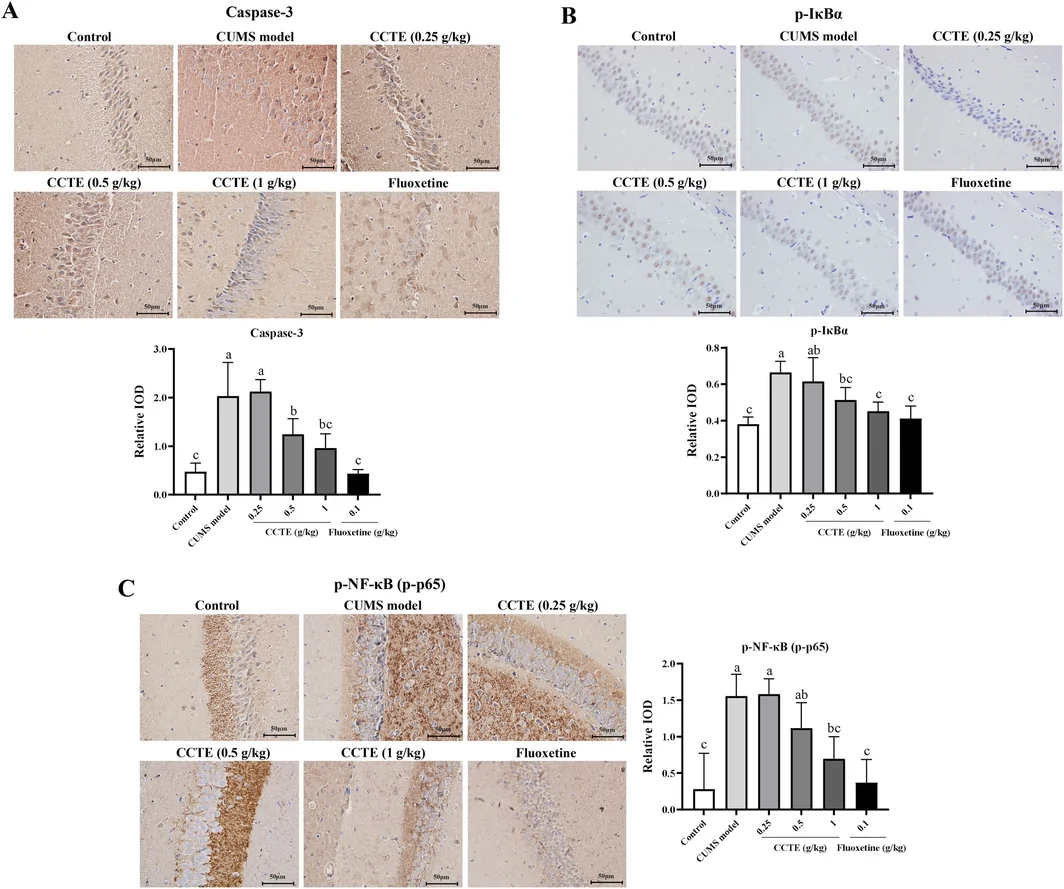
Expression of NF‐κB signaling pathway‐related proteins in hippocampal tissue. (A) Caspase‐3; (B) p‐IκBα; (C) p‐NF‐κB (p‐p65). Different letters indicate statistically significant differences (*p* < 0.05) by one‐way ANOVA followed by Tukey's post hoc test.

### 3.9 Brain Distribution of CCTE

To determine whether the major polyphenolic constituents of CCTE can traverse the blood–brain barrier (BBB) and to characterize their molecular forms in brain tissue of CUMS mice following oral administration (0.5 g/kg), we quantified these compounds in brain homogenates under three analytical conditions: (i) untreated (measuring only free aglycones), (ii) enzymatically hydrolyzed with β‐glucuronidase (to release glucuronide‐conjugated metabolites), and (iii) enzymatically hydrolyzed with sulfatase (to release sulfate‐conjugated metabolites) (Figure S2; Figure 9A–C). The LC–MS/MS analysis revealed that only three flavonoid aglycones—OKA, ISO, and TAX—were detected in the brain tissue after intragastric administration of CCTE, whereas the five flavonoid glycosides (MR, FMR, TDG, QDG, FDG) and the three phenolic acids (CA, TCA, FCA) were undetectable. Two key factors may account for this selective brain distribution profile. First, the blood–brain barrier (BBB) strictly restricts the entry of highly hydrophilic and high‐molecular‐weight compounds. Flavonoid glycosides and phenolic acids carry glucose moieties that substantially increase their polarity and molecular mass, which prevents them from crossing the intact BBB. Second, regarding the origin of the brain‐detected aglycones: CCTE intrinsically contains free OKA, ISO, and TAX (accounting for 2.0%, 3.8%, and 2.7% of the extract weight, respectively) as native constituents. However, the majority of circulating and brain‐accessible aglycones are generated via gastrointestinal deglycosylation: the parent glycosides (MR, FMR, and TDG) are hydrolyzed by intestinal glycosidases into their corresponding aglycones prior to intestinal absorption, as demonstrated in our previous rat and human pharmacokinetic studies (Han et al. 2023, 2016). These low‐molecular‐weight, moderately lipophilic aglycones are capable of traversing the BBB and distributing into the brain parenchyma. Based on the above results, it was confirmed that only OKA, ISO, and TAX, three bioactive polyphenolic substances, could be transported to the brain parenchyma after oral administration of CCTE.

**FIGURE 9:**
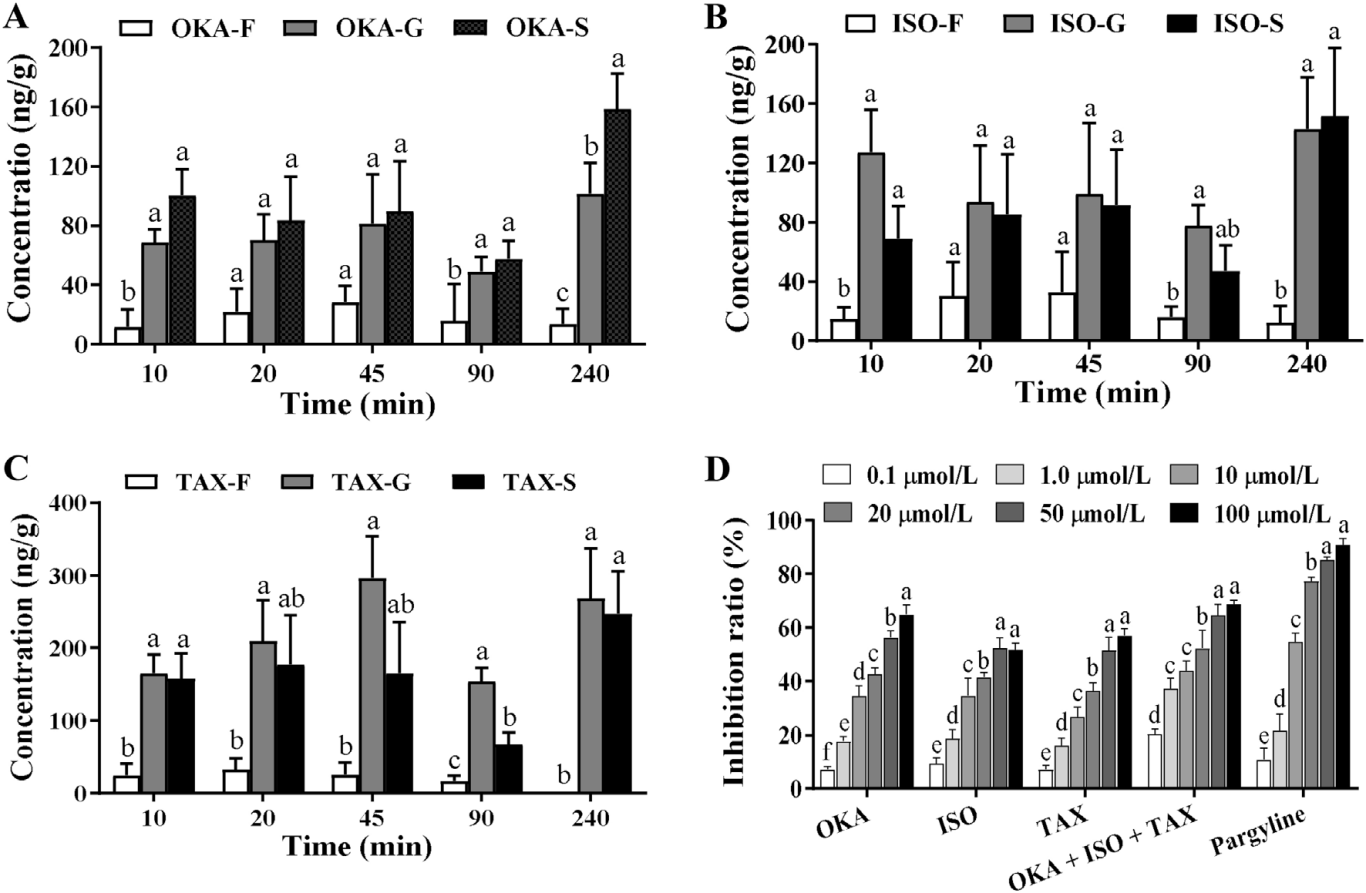
Distribution of polyphenolic compounds in the brain following oral administration of 0.5 g/kg CCTE and their inhibitory effects on MAO‐A activity. (A) OKA; (B) ISO; (C) TAX. The concentrations of OKA‐F, ISO‐F, TAX‐F were quantified in the samples untreated with β‐glucuronidase and sulfatase; the concentrations of OKA‐G, ISO‐G, TAX‐G were quantified in the samples treated with β‐glucuronidase; the concentrations of OKA‐S, ISO‐S, TAX‐S were quantified in the samples treated with sulfatase. (D) Concentration‐dependent inhibition of MAO‐A by OKA, ISO, TAX, and their equimolar mixture (1:1:1 ratio). (*n* = 6). Different letters indicate statistically significant differences (*p* < 0.05) by one‐way ANOVA followed by Tukey's post hoc test.

Concentration–time profiles revealed rapid brain uptake and extensive Phase II metabolism of all three aglycones (Figure 9A–C). OKA and ISO reached peak brain concentrations (*C*_max_) at 45 min post‐dose, whereas TAX peaked earlier—at 20 min—followed by a more rapid decline. By 240 min, TAX levels fell below the lower limit of quantification, while measurable concentrations of OKA and ISO persisted, suggesting comparatively longer brain retention for these two compounds.

In the untreated samples, *C*_max_ values for the free (unconjugated) aglycones were 28.45 ± 10.9 ng/g (OKA), 32.74 ± 27.3 ng/g (ISO), and 32.04 ± 6.9 ng/g (TAX). Following β‐glucuronidase treatment, total recoverable concentrations increased markedly: *C*_max_ rose to 81.2 ± 81.3 ng/g (OKA), 142.7 ± 84.6 ng/g (ISO), and 268.1 ± 170.3 ng/g (TAX). Sulfatase treatment similarly enhanced detection, yielding *C*_max_ values of 158.6 ± 58.8 ng/g (OKA), 151.6 ± 111.7 ng/g (ISO), and 247.2 ± 143.2 ng/g (TAX). Full quantitative datasets of brain polyphenol concentrations at all sampling time points under the three analytical conditions (untreated, β‐glucuronidase‐hydrolyzed, and sulfatase‐hydrolyzed) are summarized in Table S1; Tables S2 and S3 of the Supporting Information, respectively. Critically, the sum of aglycone concentrations after either enzymatic hydrolysis significantly exceeded those measured in untreated samples (*p* < 0.001), confirming that OKA, ISO, and TAX reside in brain tissue predominantly as glucuronide and/or sulfate conjugates.

### 3.10 Inhibition of Monoamine Oxidase A (MAO‐A) by OKA, ISO and TAX

In vitro MAO‐A inhibition assays (Figure 9D) demonstrated that the positive control drug pargyline potently suppressed MAO‐A activity, with an IC_50_ of 8.71 ± 1.21 μmol/L. The three brain‐penetrating polyphenolic aglycones—OKA, ISO and TAX—also exhibited concentration‐dependent inhibition of MAO‐A, yielding IC_50_ values of 38.66 ± 2.45 μmol/L, 48.23 ± 3.12 μmol/L, and 47.28 ± 2.98 μmol/L, respectively. OKA displayed the highest inhibitory potency among the three compounds. Critically, when combined at equimolar ratios (1:1:1), the mixture achieved markedly enhanced MAO‐A inhibition, with an IC_50_ of 18.79 ± 1.67 μmol/L—significantly lower than those of any individual component (*p* < 0.001). This robust synergistic interaction indicates that co‐administration of OKA, ISO, and TAX produces supra‐additive MAO‐A suppression. Given that MAO‐A catalyzes the oxidative deamination of 5‐HT, this synergy likely contributes to elevated synaptic 5‐HT levels in key limbic regions, thereby reinforcing the central antidepressant mechanism of CCTE.

## 4 Discussion

This study presents two core novel findings. First, it provides the first systematic elucidation of CCTE's multi‐pathway antidepressant effects in a CUMS mouse model. Unlike previous preliminary reports that only described CCTE's antidepressant‐like activity in acute behavioral despair models, this work comprehensively delineates its regulatory functions across monoaminergic transmission, HPA axis homeostasis, oxidative stress, NLRP3 inflammasome‐driven neuroinflammation, and hippocampal neuronal integrity, establishing an integrated multi‐target mechanistic framework. Second, this is the first study to identify three blood–brain barrier‐permeable flavonoid aglycones (okanin, isookanin, taxifolin) from *Coreopsis tinctoria* and confirm their synergistic MAO‐A inhibitory activity. This result clarifies the central bioactive constituents and links brain‐accessible compounds to a validated antidepressant target, defining the material basis of CCTE's central pharmacological actions. These advances set this study apart from most analogous polyphenol investigations, which typically report either behavioral efficacy without in‐depth mechanisms or in vitro activity without brain exposure evidence.

Using a CUMS‐induced mouse model of stress‐related mood disturbances, this study systematically evaluated the mood‐supporting efficacy of CCTE and characterized its mechanisms of action across five interrelated biological domains: behavioral performance, neuroendocrine homeostasis, cerebral redox balance, hippocampal neuroinflammatory signaling, and brain‐penetrant bioactive constituents. Fluoxetine was included as the in vivo positive control to provide a clinically relevant benchmark for the overall antidepressant‐like efficacy of CCTE; it was not intended to serve as a pathway‐specific comparator for MAO‐A inhibition, NF‐κB signaling, NLRP3 inflammasome activation, or oxidative stress. Accordingly, for the in vitro MAO‐A activity assay, pargyline was employed as the mechanism‐specific positive control. For the in vivo mechanistic investigations involving NF‐κB, NLRP3 inflammasome, and oxidative stress, the goal was to delineate the multi‐target profile of CCTE rather than to conduct head‐to‐head comparisons with pathway‐selective inhibitors. Behaviorally, CCTE produced dose‐dependent reversal of CUMS‐induced mood‐related deficits, including reduced sucrose preference, diminished exploratory activity in the open field test, and suppressed spontaneous locomotor activity. Notably, the high‐dose CCTE group exhibited improvements comparable to those elicited by fluoxetine, a conventional mood‐supporting agent, thereby reinforcing the robustness and practical relevance of CCTE's mood‐supporting effects. These results are consistent with established patterns in research on natural functional food ingredients for mood health (Gamage et al. 2023), wherein multicomponent plant‐based functional food ingredients—such as paeoniflorin (PF) (Lei et al. 2022) and total paeony glycosides (TPG) (Su et al. 2024)—have demonstrated efficacy in ameliorating CUMS‐induced behavioral deficits through modulation of emotion‐ and motivation‐related neural circuits.

At the mechanistic level, this study demonstrates that CCTE concurrently modulates multiple critical nodes across the depression‐related pathological network, thereby exerting a synergistic regulatory effect. Specifically, CCTE significantly increased 5‐HT levels in both peripheral and central compartments of depressed mice, decreased its primary metabolite 5‐HIAA, and elevated the 5‐HT/5‐HIAA ratio—a biochemical indicator of enhanced serotonergic neurotransmission efficiency. This modulation aligns with the classical monoamine hypothesis of depression and mirrors the pharmacological action of selective serotonin reuptake inhibitors (SSRIs) such as fluoxetine (Cooper et al. 2025; Witt et al. 2023). Nevertheless, accumulating evidence indicates that depression arises from dysregulation across interconnected biological systems—not merely isolated monoamine deficits (Cui et al. 2024). Extending beyond monoaminergic pathways, the present study further reveals that CCTE dose‐dependently normalized HPA axis hyperactivity, as evidenced by significant reductions in serum CORT, ACTH and CRH. Given that HPA axis dysregulation is a well‐validated neuroendocrine hallmark of major depressive disorder—and exhibits bidirectional crosstalk with monoaminergic circuits (Menke 2024)—this dual‐system modulation underscores CCTE's capacity for integrated pathophysiological intervention. Notably, analogous multimodal regulation has been reported for other traditional Chinese herbal formulations, including Xiao Chai Hu Tang, which similarly attenuates HPA axis overactivation (Sun et al. 2023). Collectively, these findings establish that CCTE simultaneously restores serotonergic tone and HPA axis homeostasis, offering a coherent, systems‐level mechanistic foundation for its robust mood‐supporting efficacy as a plant‐derived functional food ingredient.

A key innovative contribution of this study is the mechanistic dissection of CCTE's antidepressant action through coordinated regulation of oxidative stress and neuroinflammatory pathways—two interdependent pathological drivers in chronic stress‐induced depression. Accumulating evidence establishes that oxidative stress and neuroinflammation engage in a self‐amplifying loop, jointly promoting neuronal dysfunction and structural damage (Mehra et al. 2025; Salom et al. 2023). Consistent with this theory, CUMS disrupted hippocampal redox homeostasis with suppressed SOD and elevated MDA, consistent with canonical antioxidant impairment under chronic stress. We observed elevated CAT activity in CUMS mice, which may be a compensatory adaptive response: prolonged CUMS induces massive H_2_O_2_ accumulation, triggering CAT upregulation to offset acute oxidative damage. Following CCTE treatment, cerebral ROS overload was alleviated, eliminating the need for compensatory CAT overactivation; the recovered baseline CAT activity denotes CCTE's beneficial homeostatic regulation rather than direct CAT inhibition. In parallel, CCTE significantly elevated SOD activity and normalized the compensatory elevation of GSH toward baseline levels while lowering MDA content. These coordinated changes collectively reflect attenuation of oxidative injury via reinforcement of endogenous antioxidant capacity and suppression of lipid peroxidation. This redox‐modulating profile parallels the established antidepressant mechanisms of bioactive polyphenols—including magnolol (Cheng et al. 2018) and curcumin (Hussain et al. 2022).

In terms of peripheral systemic inflammation, CCTE exerted a dose‐dependent suppressive effect on circulating pro‐inflammatory mediators. Specifically, medium‐dose CCTE significantly reduced serum levels of TNF‐α and IL‐1β, while high‐dose CCTE significantly decreased all four measured markers: TNF‐α, IL‐6, IL‐1β, and NF‐κB. This peripheral anti‐inflammatory activity aligns with the well‐documented immunomodulatory properties of plant‐derived polyphenols, which mitigate chronic stress–induced systemic low‐grade inflammation. Notably, the anti‐inflammatory efficacy of high‐dose CCTE in the peripheral circulation was comparable to that of fluoxetine, indicating its favorable capacity to restore peripheral immune homeostasis under chronic stress. With respect to central hippocampal neuroinflammation, our immunohistochemical results demonstrated that CCTE directly inhibited NLRP3 inflammasome activation in the hippocampal CA1 region in a dose‐dependent manner, as evidenced by downregulated protein expression of NLRP3, ASC, and cleaved Caspase‐1. In parallel, CCTE suppressed the upstream canonical NF‐κB signaling cascade, as reflected by decreased phosphorylation of NF‐κB p65 and IκBα, which further reduced the expression of the neuronal apoptosis marker cleaved Caspase‐3. The hippocampal NLRP3/NF‐κB axis constitutes a pivotal pathogenic hub linking chronic stress, neuroinflammation, and depressive pathophysiology; its overactivation propagates central inflammatory cascades, impairs synaptic plasticity, and disrupts monoaminergic neurotransmission and HPA axis feedback (Wang et al. 2025, 2024). Similar pathway‐specific inhibition of central neuroinflammation has also been reported for other natural bioactive compounds such as paeoniflorin (Wang et al. 2024) and astragaloside IV (Song et al. 2018). This study provides the first experimental evidence that CCTE concurrently targets both NLRP3 inflammasome assembly and canonical NF‐κB signal transduction in the hippocampus, directly blocking the central inflammatory cascade that drives neuronal damage and depressive‐like behaviors. Functionally, the peripheral and central anti‐inflammatory effects of CCTE act in a coordinated manner. Peripheral cytokine reduction alleviates inflammatory signals that reach the brain via the circulation, while direct hippocampal NLRP3/NF‐κB inhibition interrupts the central inflammatory amplification loop. Together, these two layers of regulation constitute the multi‐compartmental anti‐inflammatory mechanism underlying the antidepressant effect of CCTE (Zhang et al. 2026).

Importantly, IL‐1β upregulates serotonin transporter (SERT) expression to accelerate 5‐HT reuptake and reduce serotonergic neurotransmission (Chang et al. 2024; Jiang, Yao, et al. 2024). Beyond serotonergic regulation, NF‐κB/NLRP3‐driven pro‐inflammatory cytokines (TNF‐α, IL‐6, IL‐1β) act as critical upstream modulators of HPA axis hyperactivity: they directly stimulate hypothalamic CRH secretion, promote pituitary ACTH release, and impair glucocorticoid negative feedback, driving the sustained HPA overactivation observed in our CUMS model. In turn, chronically elevated corticosterone further amplifies hippocampal oxidative stress and NF‐κB activity, forming a self‐sustaining positive feedback loop between neuroinflammation and HPA dysregulation. Meanwhile, hypercortisolemia upregulates MAO‐A expression to accelerate 5‐HT catabolism, creating an additional cascade linking HPA dysfunction to monoamine deficiency (Wang et al. 2019; Yan et al. 2022). Collectively, these interactions form an integrated network centered on the NLRP3/NF‐κB inflammatory axis: chronic stress initiates hippocampal NLRP3 assembly and NF‐κB activation as the upstream pathogenic event, which induces HPA hyperactivity and serotonergic dysfunction as two interlinked downstream branches that mutually reinforce each other (Ao et al. 2025; Wang et al. 2025). In this network, CCTE exerts multi‐node regulation: it suppresses the core NLRP3/NF‐κB cascade to interrupt upstream inflammatory drive, and its brain‐penetrant aglycones (OKA, ISO, TAX) directly and synergistically inhibit MAO‐A to reduce 5‐HT degradation. Such concurrent modulation of the NLRP3/NF‐κB inflammatory cascade and serotonergic catabolism collectively contributes to the amelioration of depressive‐like behaviors and hippocampal neuronal damage in CUMS‐exposed mice.

Elucidating the brain distribution of active constituents is a prerequisite for interpreting the central mechanism of action of natural products. Due to the typically high polarity of most plant polyphenols, which limits their ability to cross the blood–brain barrier (BBB), research into their central nervous system efficacy is often constrained (Zhang et al. 2021). This study, for the first time, employed LC–MS/MS analysis to confirm that only three flavonoid aglycones from CCTE—OKA, ISO and TAX—entered brain tissue. Their relatively low polarity and molecular weight likely constitute key factors for BBB penetration (Carecho et al. 2024). The time to peak concentration was approximately 45 min, and the compounds remained detectable at 240 min, indicating favorable brain retention. Of note, brain concentrations showed relatively high inter‐individual variability, which is consistent with the known pharmacokinetic characteristics of orally administered polyphenols and may be further amplified by physiological heterogeneity introduced by chronic stress. These findings not only provide pharmacokinetic support for the observed effects of CCTE on central monoamine neurotransmitters, oxidative stress, and neuroinflammation but also align with the reported brain distribution characteristics of certain other flavonoid compounds (Ambrogini et al. 2015; Reddy et al. 2020). Further investigation demonstrated that OKA, ISO and TAX each inhibit MAO‐A in a concentration‐dependent manner, with IC₅₀ values of 38.66 ± 2.45, 48.23 ± 3.12, and 47.28 ± 2.98 μmol/L, respectively—OKA exhibiting the highest potency. Although their inhibitory activities are lower than that of the reference inhibitor pargyline (IC₅₀ = 8.71 μmol/L), this study provides the first direct experimental evidence that these three flavonoid aglycones engage MAO‐A—a well‐established therapeutic target in depression. These findings support a mechanistic model wherein the three BBB‐permeable flavonoid aglycones exert combined MAO‐A inhibitory effects, consistent with the polypharmacological properties typically observed for multicomponent natural product extracts. It should be noted that the peak total concentrations measured in whole‐brain homogenate are lower than the in vitro IC₅₀ values. However, this comparison is based on bulk tissue concentrations, whereas MAO‐A is concentrated in mitochondrial compartments of presynaptic terminals where lipophilic aglycones may accumulate to higher local levels. When considered together with the potential synergistic effects of the three aglycones, as well as their additional contributions from anti‐inflammatory, antioxidant, and HPA‐regulating effects, MAO‐A inhibition likely acts as one component of the integrated multi‐target mechanism underlying the central efficacy of CCTE, aligning with the observed elevation of cerebral 5‐HT levels.

It should be noted that this study only verified the synergistic inhibition of MAO‐A by these three compounds in vitro. Whether this effect is maintained in the complex in vivo environment, as well as the specific molecular mechanisms underlying the synergy (e.g., selectivity for MAO‐A subtypes, impacts on metabolic stability), require further elucidation through integrated in vivo pharmacokinetic‐pharmacodynamic studies and molecular docking approaches (Fan et al. 2023; Xiao et al. 2024). This study also has several limitations. First, while the CUMS model can simulate core features of depression, a gap remains between the model and the heterogeneity of human disease. Future studies could employ models more closely aligned with clinical conditions, such as social defeat stress, for further validation (Antoniuk et al. 2019). Second, CCTE is a complex mixture. This study focused only on three brain‐penetrating aglycones; other constituents may contribute indirectly to the antidepressant effect via peripheral mechanisms. The specific roles and interaction networks of all components need to be further resolved through activity‐guided fractionation and network pharmacology. Third, the mechanistic investigation primarily concentrated on the hippocampus and did not comprehensively examine other depression‐related brain regions (Jiang, Noble, et al. 2024). Fourth, the conclusions were largely based on correlative in vivo evidence and lacked direct functional validation of key targets (e.g., NLRP3) at the cellular and molecular levels. Fifth, from a food nutrition perspective, this study only evaluated the overall efficacy and mechanism of the extract in animal models, and did not carry out key nutrition‐related experiments including in vitro simulated gastrointestinal digestion, systematic bioavailability assessment, raw material safety detection, and human dietary equivalent dose conversion. These contents are important for the subsequent development and application of CCTE as a functional food ingredient, and need to be systematically addressed in follow‐up studies. Based on these considerations, future research could: (1) Systematically identify the key bioactive food constituents in CCTE and clarify their synergistic relationships by combining activity‐guided separation with network pharmacology; (2) Utilize molecular docking simulations, cellular models, and gene‐editing technologies to elucidate the binding modes, structure–activity relationships, and direct actions of monomers such as OKA, ISO, and TAX on MAO‐A, the NLRP3/NF‐κB pathway, and neurotrophic factors (e.g., BDNF); (3) Employ in vivo neural circuit techniques to explore the modulatory effects of CCTE on mood health‐related neural circuits; (4) Conduct systematic raw material safety detection and pharmacokinetic studies to define the safe application range, metabolic pathways, and potential toxicity of CCTE, thereby providing a basis for its development as a natural functional food ingredient for mood support.

## 5 Conclusions

In conclusion, this study confirms that CCTE exerts definite mood‐supporting effects in the CUMS‐induced stress‐related mood disturbance model. Its functional mechanisms involve multiple interrelated regulatory pathways, including modulation of monoamine neurotransmitters, suppression of HPA axis hyperactivity, mitigation of oxidative stress, and alleviation of neuroinflammation via inhibition of the NLRP3/NF‐κB signaling pathway. For the first time, it was demonstrated that the three flavonoid aglycones—OKA, ISO, and TAX—penetrate the blood–brain barrier and synergistically inhibit MAO‐A, providing a solid functional material basis and target‐specific evidence for the central mood‐supporting effects of CCTE. These findings deepen the understanding of the scientific basis underlying the mood‐regulating action of *C. tinctoria* and offer experimental support for the development of *C. tinctoria* ‐based natural functional food ingredients for mood health support.

## Author Contributions

**Haixia Han:** conceptualization, supervision, funding acquisition, writing – review and editing, writing – original draft. **Yujing Feng:** conceptualization, data curation, writing – original draft. **Zhen Wang:** data curation, methodology. **Rui Ma:** data curation, writing – original draft. **Jingjing Sun:** methodology, data curation. **Xiuli Yang:** visualization, data curation. **Yue Cui:** data curation, formal analysis. **Juan Wang:** methodology, writing – review and editing. **Mingxi Jia:** data curation, funding acquisition, writing – original draft, writing – review and editing.

## Funding

This work was supported by the Xinjiang Uygur Autonomous Region Tianshan Talent Science and Technology Innovation Team Project “Xinjiang Special Fruits and Vegetables Green, Low‐Carbon Processing Technology System Innovation Team” (2023TSYCTD0005); National Natural Science Foundation of China (81760777); Natural Science Foundation of Xinjiang Uygur Autonomous Region (2025D01B56); Xinjiang Uygur Autonomous Region “Tianchi Talent” Introduction Program Project (2225ZZQRCXM).

## Ethics Statement

The animal study protocol was approved by the Ethics Committee of Xinjiang Agricultural University Laboratory Animal Centre (approval No. 2021100). All experimental procedures and animal care were performed in strict accordance with the Guidelines for the Ethical Review of Laboratory Animal Welfare (People's Republic of China National Standard GB/T 35892‐2018), as well as the Animal Ethics Procedures and Guidelines of the Ministry of Agriculture and Rural Affairs of China. Additionally, the reporting of this animal study fully complies with the ARRIVE (Animal Research: Reporting of In Vivo Experiments) guidelines to ensure transparent and complete reporting of our in vivo research.

## Conflicts of Interest

The authors declare no conflicts of interest.

## Supporting information


**Figure S1:** A structural diagram of the mouse hippocampus tissue.
**Figure S2:** Representative chromatograms of OKA, ISO, and TAX in mouse brain tissue.
**Table S1:** Determination of OKA levels in brain tissue from mice following oral administration of CCTE.
**Table S2:** Determination of ISO levels in brain tissue from mice following oral administration of CCTE.
**Table S3:** Determination of TAX levels in brain tissue from mice following oral administration of CCTE.

## Data Availability

The authors confirm that the data supporting the findings of this study are available within the article and Supporting Information. All data generated or analyzed in the current study are available from the corresponding author on reasonable request.

## References

[fsn372480-bib-0001] Ambrogini, P. , D. Grande , L. Gennari , et al. 2015. “Enhancement of Flavonoid Ability to Cross the Blood–Brain Barrier of Rats by Co‐Administration With α‐Tocopherol.” Food & Function 6: 394–400. 10.1039/c4fo00817k. 25474041

[fsn372480-bib-0002] Antoniuk, S. , M. Bijata , E. Ponimaskin , and J. Wlodarczyk . 2019. “Chronic Unpredictable Mild Stress for Modeling Depression in Rodents: Meta‐Analysis of Model Reliability.” Neuroscience and Biobehavioral Reviews 99: 101–116. 10.1016/j.neubiorev.2018.12.002. 30529362

[fsn372480-bib-0003] Ao, W. , W. Gao , and T. Li . 2025. “Research Progress on the Mechanism of Antidepressant Effect of Salidroside.” International Immunopharmacology 162: 115205. 10.1016/j.intimp.2025.115205. 40651432

[fsn372480-bib-0004] Behl, T. , T. Rana , G. H. Alotaibi , et al. 2022. “Polyphenols Inhibiting MAPK Signalling Pathway Mediated Oxidative Stress and Inflammation in Depression.” Biomedicine & Pharmacotherapy 146: 112545. 10.1016/j.biopha.2021.112545. 34922112

[fsn372480-bib-0005] Carecho, R. , D. Marques , D. Carregosa , et al. 2024. “Circulating Low‐Molecular‐Weight (Poly)phenol Metabolites in the Brain: Unveiling In Vitro and In Vivo Blood–Brain Barrier Transport.” Food & Function 15: 7812–7827. 10.1039/D4FO01396D. 38967492

[fsn372480-bib-0006] Chang, J. , H. Yang , X. Shan , et al. 2024. “Bergamot Essential Oil Improves CUMS‐Induced Depression‐Like Behaviour in Rats by Protecting the Plasticity of Hippocampal Neurons.” Journal of Cellular and Molecular Medicine 28: e18178. 10.1111/jcmm.18178. PMC10980931 38553964

[fsn372480-bib-0007] Cheng, J. , S. Dong , L. Yi , D. Geng , and Q. Liu . 2018. “International Immunopharmacology Magnolol Abrogates Chronic Mild Stress‐Induced Depressive‐Like Behaviors by Inhibiting Neuroinflammation and Oxidative Stress in the Prefrontal Cortex of Mice.” International Immunopharmacology 59: 61–67. 10.1016/j.intimp.2018.03.031. 29627576

[fsn372480-bib-0008] Cooper, D. G. , J. P. Cowden , and P. M. Vo . 2025. “Gene Expression Signatures of Response to Fluoxetine Treatment: Systematic Review and Meta‐Analyses.” Molecular Psychiatry 30: 5563–5577. 10.1038/s41380-025-03118-6. PMC12532588 40676137

[fsn372480-bib-0009] Cui, L. , S. Li , S. Wang , et al. 2024. “Major Depressive Disorder: Hypothesis, Mechanism, Prevention and Treatment.” Signal Transduction and Targeted Therapy 9: 30. 10.1038/s41392-024-01738-y. PMC10853571 38331979

[fsn372480-bib-0010] Ding, W. , L. Wang , L. Li , et al. 2024. “Pathogenesis of Depression and the Potential for Traditional Chinese Medicine Treatment.” Frontiers in Pharmacology 15: 1407869. 10.3389/fphar.2024.1407869. PMC11231087 38983910

[fsn372480-bib-0011] Duman, R. S. , G. K. Aghajanian , G. Sanacora , J. H. Krystal , and N. Haven . 2016. “Synaptic Plasticity and Depression: New Insights From Stress and Rapid‐Acting Antidepressants.” Nature Medicine 22: 238–249. 10.1038/nm.4050.Synaptic. PMC5405628 26937618

[fsn372480-bib-0012] Fan, L. , Y. Peng , and X. Li . 2023. “Phytomedicine Brain Regional Pharmacokinetics of Hydroxytyrosol and Its Molecular Mechanism Against Depression Assessed by Multi‐Omics Approaches.” Phytomedicine 112: 154712. 10.1016/j.phymed.2023.154712. 36774845

[fsn372480-bib-0013] Gamage, E. , R. Orr , N. Travica , et al. 2023. “Polyphenols as Novel Interventions for Depression: Exploring the Efficacy, Mechanisms of Action, and Implications for Future Research.” Neuroscience and Biobehavioral Reviews 151: 105225. 10.1016/j.neubiorev.2023.105225. 37164045

[fsn372480-bib-0014] Han, H. , R. Ma , A. Xie , et al. 2023. “Development of an LC/MS/MS Method for Simultaneous Detection of 11 Polyphenols in Rat Plasma and Its Pharmacokinetic Application After Oral Administration of *Coreopsis tinctoria* Extract.” Chemistry & Biodiversity 19: e202200574. 10.1002/cbdv.202200574. 36382445

[fsn372480-bib-0015] Han, H. , Z. Ma , W. Wang , et al. 2016. “Deglycosylation and Absorption of Marein, Flavanomarein and Taxifolin‐7‐O‐β‐D‐Glucopyranoside From Capitula of *Coreopsis tinctoria* in Rats and Humans.” Journal of Functional Foods 27: 178–188. 10.1016/j.jff.2016.09.005.

[fsn372480-bib-0016] Hussain, H. , S. Ahmad , S. Wadood , et al. 2022. “Investigation of Antistress and Antidepressant Activities of Synthetic Curcumin Analogues: Behavioral and Biomarker Approach.” Biomedicine 10: 2385. 10.3390/biomedicines10102385. PMC9598253 36289646

[fsn372480-bib-0017] Jiang, N. , C. Yao , Y. Zhang , et al. 2024. “Antidepressant Effects of Parishin C in Chronic Social Defeat Stress‐Induced Depressive Mice.” Journal of Ethnopharmacology 325: 117891. 10.1016/j.jep.2024.117891. 38331122

[fsn372480-bib-0018] Jiang, R. , S. Noble , M. Rosenblatt , et al. 2024. “The Brain Structure, Inflammatory, and Genetic Mechanisms Mediate the Association Between Physical Frailty and Depression.” Nature Communications 15: 4411. 10.1038/s41467-024-48827-8. PMC11116547 38782943

[fsn372480-bib-0019] Lei, C. , Z. Chen , L. Fan , et al. 2022. “Integrating Metabolomics and Network Analysis for Exploring the Mechanism Underlying the Antidepressant Activity of Paeoniflorin in Rats With CUMS‐Induced Depression.” Frontiers in Pharmacology 13: 904190. 10.3389/fphar.2022.904190. PMC9234202 35770096

[fsn372480-bib-0020] Ma, R. , S. Li , Z. Wang , J. Gao , A. Xie , and H. Han . 2022. “Screening of Effective Anti‐Depressant Part From Coreopsis Tinctoria and Study on Its Mechanism.” China Pharmacy 33: 1177–1182. 10.6039/j.issn.1001-0408.2022.10.05.

[fsn372480-bib-0021] Ma, Z. , S. Zheng , H. Han , et al. 2016. “The Bioactive Components of *Coreopsis tinctoria* (Asteraceae) Capitula: Antioxidant Activity In Vitro and Profile in Rat Plasma.” Journal of Functional Foods 20: 575–586. 10.1016/j.jff.2015.11.023.

[fsn372480-bib-0022] Mehra, A. , J. Khanna , G. Singh , V. Sachdeva , and N. Bedi . 2025. “A Comprehensive Review on Major Depressive Disorder: Exploring Etiology, Pathogenesis and Clinical Approaches.” Current Behavioral Neuroscience Reports 12: 18. 10.1007/s40473-025-00308-y.

[fsn372480-bib-0023] Menke, A. 2024. “The HPA Axis as Target for Depression.” Current Neuropharmacology 22: 904–915. 10.2174/1570159X21666230811141557. PMC10845091 37581323

[fsn372480-bib-0024] Mi, Y. , J. Xu , R. Shi , et al. 2023. “Okanin From *Coreopsis tinctoria* Nutt. Alleviates Cognitive Impairment in Bilateral Common Carotid Artery Occlusion Mice by Regulating the miR‐7/NLRP3 Axis in Microglia.” Food & Function 14: 369–387. 10.1039/d2fo01476a. 36511396

[fsn372480-bib-0025] Noori, T. , A. Sureda , and S. Shirooie . 2022. “The Role of Natural Products in Treatment of Depressive Disorder.” Current Neuropharmacology 20: 929–949. 10.2174/1570159X20666220103140834. PMC9881107 34979889

[fsn372480-bib-0026] Reddy, V. P. , P. Aryal , S. Robinson , R. Rafiu , M. Obrenovich , and G. Perry . 2020. “Polyphenols in Alzheimer's Disease and in the Gut‐Brain Axis.” Microorganisms 8: 199. 10.3390/microorganisms8020199. PMC7074796 32023969

[fsn372480-bib-0027] Rog, J. , P. Pawlikowska , M. Futyma‐j , et al. 2026. “Polyphenol Consumption and Its Association With Physical and Mental Health in Adults With Major Depressive Disorder.” Nutrients 18: 47. 10.3390/nu18010047. PMC12788040 41515165

[fsn372480-bib-0028] Salom, A. , A. Cardoso , and N. Vale . 2023. “Oxidative Stress in Depression: The Link With the Stress Response, Neuroinflammation, Serotonin, Neurogenesis and Synaptic Plasticity.” Antioxidants 12: 470. 10.3390/antiox12020470. PMC9951986 36830028

[fsn372480-bib-0029] Song, M. , J. Ruan , R. Zhang , J. Deng , Z. Ma , and S. Ma . 2018. “Astragaloside IV Ameliorates Neuroinflammation‐Induced Depressive‐Like Behaviors in Mice via the PPARγ/NF‐κB/NLRP3 Inflammasome Axis.” Acta Pharmacologica Sinica 39: 1559–1570. 10.1038/aps.2017.208. PMC6289360 29795356

[fsn372480-bib-0030] Su, L. , H. Lu , D. Zhang , et al. 2024. “Total Paeony Glycoside Relieves Neuroinflammation to Exert Antidepressant Effect via the Interplay Between NLRP3 Inflammasome, Pyroptosis and Autophagy.” Phytomedicine 128: 155519. 10.1016/j.phymed.2024.155519. 38492365

[fsn372480-bib-0031] Sun, C. , M. Gao , and M. Qiao . 2023. “Research Progress of Traditional Chinese Medicine Compound “Xiaochaihu Decoction” in the Treatment of Depression.” Biomedicine & Pharmacotherapy 159: 114249. 10.1016/j.biopha.2023.114249. 36682244

[fsn372480-bib-0032] Wang, H. , Z. Wang , and N. Chen . 2021. “The Receptor Hypothesis and the Pathogenesis of Depression: Genetic Bases and Biological Correlates.” Pharmacological Research 167: 105542. 10.1016/j.phrs.2021.105542. 33711432

[fsn372480-bib-0033] Wang, J. , C. Cheng , C. Xin , and Z. Wang . 2019. “The Antidepressant‐Like Effect of Flavonoids From *Trigonella Foenum‐graecum* Seeds in Chronic Restraint Stress Mice via Modulation of Monoamine Regulatory Pathways.” Molecules 24: 1105. 10.3390/molecules24061105. PMC6471463 30897781

[fsn372480-bib-0034] Wang, L. , H. Li , Y. Zhao , et al. 2025. “Artemisinin Alleviates Chronic Social Defeat Stress‐Induced Depressive‐Like Behavior by Inhibiting Neuroinflammation via the TLR4/MyD88/NF‐κB/NLRP3 Signaling Pathway.” Phytotherapy Research 0: 1–19. 10.1002/ptr.70106. 41053956

[fsn372480-bib-0035] Wang, X. , L. Su , S. Liu , et al. 2024. “Paeoniflorin Inhibits the Activation of Microglia and Alleviates Depressive Behavior by Regulating SIRT1‐NF‐kB‐NLRP3/Pyroptosis Pathway.” International Journal of Molecular Sciences 25: 12543. 10.3390/ijms252312543. PMC11640976 39684254

[fsn372480-bib-0036] Wang, Z. , M. Jia , N. Yadikar , et al. 2026. “ *Coreopsis tinctoria* Nutt. Polyphenols Protect Intestinal Barrier in Depressed Mice by Modulating TLR4/MYD88/NF‐κB Pathway.” Chemistry & Biodiversity 23: e03020. 10.1002/cbdv.202503020. 41707058

[fsn372480-bib-0037] Witt, C. E. , S. Mena , J. Holmes , et al. 2023. “Article Serotonin Is a Common Thread Linking Different Classes of Antidepressants Ll Ll Article Serotonin Is a Common Thread Linking Different Classes of Antidepressants.” Cell Chemical Biology 30: 1557–1570. 10.1016/j.chembiol.2023.10.009. 37992715

[fsn372480-bib-0038] Xiao, F. , Y. Song , G. Wang , and J. Aa . 2024. “Intestinal Metabolism of Crocin and a Pharmacokinetics and Pharmacodynamics Study in the Chronic Social Defeat Stress Mouse Model.” Pharmaceuticals 17: 843. 10.3390/ph17070843. PMC11279738 39065694

[fsn372480-bib-0039] Yan, L. , J. Wang , X. He , et al. 2022. “ *Platycladus orientalis* Seed Extract as a Potential Triple Reuptake MAO Inhibitor Rescue Depression Phenotype Through Restoring Monoamine Neurotransmitters.” Journal of Ethnopharmacology 295: 115302. 10.1016/j.jep.2022.115302. 35489661

[fsn372480-bib-0040] Yin, Y. , T. Ju , D. Zeng , et al. 2024. ““Inflamed” Depression: A Review of the Interactions Between Depression and Inflammation and Current Anti‐Inflammatory Strategies for Depression.” Pharmacological Research 207: 107322. 10.1016/j.phrs.2024.107322. 39038630

[fsn372480-bib-0041] Zhang, X. , H. Xue , C. Zhu , and C. Ji . 2026. “Neuroinflammatory Mechanisms and Pharmacological Advances in Autism Spectrum Disorder: From Inflammatory Pathways to Targeted Interventions.” Frontiers in Immunology 17: 1829127. 10.3389/fimmu.2026.1829127. PMC13229736 42245635

[fsn372480-bib-0042] Zhang, Y. , C. Lv , and G. Zhao . 2021. “Ways to Enhance the Bioavailability of Polyphenols in the Brain: A Journey Through the Blood‐Brain Barrier.” Food Reviews International 38: 1888973. 10.1080/87559129.2021.1888973.

