# Supplementary material for: Coreopsis tinctoria Nutt. Polyphenols Ameliorate CUMS‐Induced Depressive Behaviors and Hippocampal Neuronal Damage in Mice
Source: Food Sci Nutr. 2026 Oct 16;14(10):e72480. doi: 10.1002/fsn3.72480 (PMC13674004; doi:10.1002/fsn3.72480)
Supplement: Supplementary file 1 [file FSN3-14-e72480-s001.docx]

Supplementary Materials

**Figure S1.** A structural diagram of the mouse hippocampus tissue.

**Figure S2.** Representative chromatograms of OKA, ISO, and TAX in mouse brain tissue. (A) Blank brain tissue with no test substances or internal standards added; (B) Blank brain tissue with the target test substance (30 ng/mL) added; (C) Brain tissue sample collected 0.5 hours after oral administration of CCTE to mice.

**Table S1.** Determination of OKA levels in brain tissue from mice following oral administration of CCTE.

|  | OKA-F (ng/g) | | | | | | OKA-G (ng/g) | | | | | | OKA-S (ng/g) | | | | | |
| --- | --- | --- | --- | --- | --- | --- | --- | --- | --- | --- | --- | --- | --- | --- | --- | --- | --- | --- |
| 10 min | 5.61 | 35.09 | 8.33 | 9.01 | 5.42 | 6.54 | 65.05 | 66.64 | 48.85 | 110.54 | 60.74 | 60.19 | 63.91 | 158.27 | 73.29 | 154.17 | 74.23 | 78.48 |
| 20 min | 6.87 | 21.93 | 11.05 | 45.16 | 35.51 | 8.67 | 49.57 | 131.32 | 51.40 | 27.59 | 43.80 | 117.42 | 72.47 | 301.38 | 29.64 | 8.12 | 54.88 | 234.46 |
| 45 min | 21.24 | 31.82 | 13.91 | 28.90 | 46.45 | 28.36 | 45.48 | 15.39 | 229.62 | 61.39 | 19.19 | 115.88 | 78.32 | 33.99 | 255.05 | 64.77 | 46.49 | 58.93 |
| 90 min | 5.26 | 5.03 | 6.73 | 4.89 | 6.39 | 66.34 | 62.51 | 16.81 | 59.66 | 66.45 | 68.70 | 19.19 | 72.22 | 29.44 | 83.34 | 45.20 | 93.83 | 21.29 |
| 240 min | 10.79 | 5.35 | 10.23 | 33.24 | 15.59 | 6.04 | 56.31 | 70.93 | 168.88 | 47.04 | 148.24 | 117.92 | 157.16 | 241.07 | 136.06 | 69.44 | 201.79 | 145.89 |

**Table S2.** Determination of ISO levels in brain tissue from mice following oral administration of CCTE.

|  | ISO-F (ng/g) | | | | | | ISO-G (ng/g) | | | | | | ISO-S (ng/g) | | | | | |
| --- | --- | --- | --- | --- | --- | --- | --- | --- | --- | --- | --- | --- | --- | --- | --- | --- | --- | --- |
| 10 min | 5.24 | 26.84 | 10.58 | 15.71 | 8.25 | 20.59 | 89.75 | 123.55 | 247.49 | 167.73 | 76.76 | 57.91 | 25.01 | 146.17 | 34.20 | 128.27 | 41.87 | 37.68 |
| 20 min | 23.10 | 71.52 | 22.38 | 40.94 | 9.57 | 13.37 | 77.30 | 236.78 | 19.13 | 14.17 | 35.21 | 180.32 | 30.87 | 195.75 | 17.84 | 27.28 | 9.57 | 230.32 |
| 45 min | 14.75 | 54.13 | 21.89 | 10.28 | 78.42 | 16.98 | 88.36 | 17.74 | 320.04 | 125.02 | 32.63 | 11.01 | 30.31 | 129.99 | 259.11 | 51.16 | 64.73 | 13.26 |
| 90 min | 22.64 | 23.41 | 6.02 | 13.99 | 9.26 | 20.39 | 57.88 | 38.56 | 76.03 | 124.96 | 111.64 | 56.73 | 10.52 | 123.13 | 19.78 | 38.38 | 68.53 | 22.55 |
| 240 min | 2.93 | 7.42 | 6.56 | 14.83 | 33.83 | 7.71 | 70.33 | 82.57 | 179.25 | 127.19 | 296.38 | 100.93 | 232.73 | 263.42 | 66.47 | 41.17 | 262.38 | 43.80 |

**Table S3.** Determination of TAX levels in brain tissue from mice following oral administration of CCTE.

|  | TAX-F (ng/g) | | | | | | TAX-G (ng/g) | | | | | | TAX-S (ng/g) | | | | | |
| --- | --- | --- | --- | --- | --- | --- | --- | --- | --- | --- | --- | --- | --- | --- | --- | --- | --- | --- |
| 10 min | 1.66 | 105.23 | 12.98 | 10.56 | 7.66 | 10.87 | 125.3 | 285.66 | 186.84 | 130.59 | 140.91 | 121.17 | 83.95 | 285.40 | 129.32 | 244.92 | 88.81 | 115.70 |
| 20 min | 18.01 | 26.79 | 112.4 | 12.78 | 18.56 | 3.66 | 183.7 | 340.69 | 88.96 | 92.20 | 102.35 | 341.01 | 147.11 | 438.1 | 61.05 | 70.48 | 24.55 | 322.90 |
| 45 min | 8.99 | 6.55 | 15.3 | 1.55 | 109.7 | 10.77 | 170.7 | 138.03 | 462.88 | 225.63 | 336.03 | 445.76 | 93.424 | 92.75 | 513.45 | 136.78 | 105.25 | 51.022 |
| 90 min | 6.77 | 48.77 | 5.88 | 10.99 | 23.66 | 5.77 | 104.0 | 106.62 | 150.12 | 194.79 | 198.10 | 122.74 | 81.96 | 39.79 | 98.80 | 27.68 | 125.85 | 29.11 |
| 240 min | 6.89 | 2.91 | 5.67 | 7.89 | 6.35 | 6.99 | 118.1 | 169.97 | 335.68 | 218.04 | 582.51 | 183.84 | 300.35 | 315.77 | 152.75 | 139.89 | 476.21 | 98.44 |
